# Bridging subjective and neural state transitions in the rubber hand illusion: a neurophenomenological study

**DOI:** 10.1093/nc/niag040

**Published:** 2026-07-29

**Authors:** Yuki Tsuji, Miyuki Azuma, Camille Lépingle, Momoka Kimuro, Mika Ishizu, Fugo Suzuki, Yves Rossetti, Sotaro Shimada

**Affiliations:** School of Systems Information Science, Future University Hakodate, 116-2 Kamedanakano-cho, Hakodate, Hokkaido, 041-8655, Japan; Organization for the Strategic Coordination of Research and Intellectual Properties, Meiji University, 1-1-1 Higashi-mita, Tama-ku, Kawasaki, Kanagawa, 214-8571, Japan; Department of Human Sciences, École Normale Supérieure de Lyon, 15 parvis René Descartes, BP 7000, 69342 Lyon Cedex 07, Lyon, France; Graduate School of Science and Technology, Meiji University, 1-1-1 Higashi-mita, Tama-ku, Kawasaki, Kanagawa, 214-8571, Japan; Graduate School of Science and Technology, Meiji University, 1-1-1 Higashi-mita, Tama-ku, Kawasaki, Kanagawa, 214-8571, Japan; Graduate School of Science and Technology, Meiji University, 1-1-1 Higashi-mita, Tama-ku, Kawasaki, Kanagawa, 214-8571, Japan; Inserm Unité Mixte de Recherche-Santé (UMR-S) 1028, CNRS UMR 5292, Trajectoires team, Centre de Recherche en Neurosciences de Lyon, Université Claude Bernard Lyon 1, 16 Avenue du Doyen Lépine, 69500 Bron, France; School of Science and Technology, Meiji University, 1-1-1 Higashi-mita, Tama-ku, Kawasaki, Kanagawa, 214-8571, Japan

**Keywords:** body ownership, rubber hand illusion, neurophenomenology, EEG, graph neural networks, optimal transport

## Abstract

The rubber hand illusion (RHI) provides a powerful paradigm for probing the malleability of bodily self-consciousness. While conventional studies rely on proprioceptive drift, questionnaires, or averaged neural measures, phenomenological research suggests that the RHI unfolds through multiple subjective state transitions rather than as a simple binary phenomenon. Participants often describe ambiguous coexistence of real and artificial hands, partial incorporation, and eventual ownership, indicating meaningful dynamics of selfhood. Here, we adopt a neurophenomenological approach that integrates first-person interviews with electroencephalographic (EEG) functional connectivity network analysis. Subjective trajectories during the RHI were characterized through phenomenological interviews and modeled as successive states. EEG connectivity was analyzed using graph neural networks, enabling data-driven clustering of neural states aligned with subjective transitions. To establish a correspondence between the phenomenological and neural domains, we employed Gromov–Wasserstein (GW) optimal transport, which maps structural correspondences between subjective and EEG-derived state spaces despite their differing metrics. The results show that specific subjective transitions correspond to distinct changes in network topology. Major subjective states were associated with reconfigurations of connectivity patterns and shifts in degree centrality across several regions, including the premotor cortex and insula, which are known to contribute to body ownership. GW optimal transport further revealed a principled correspondence between the geometry of subjective and neural state spaces. Together, these findings demonstrate that subjective transitions in the RHI are mirrored by dynamic reorganization of brain networks, illustrating how neurophenomenology combined with computational modeling can advance the study of embodied selfhood.

## Introduction

The sense of bodily self is a fundamental dimension of human consciousness, integrating perceptual, motor, and affective processes into the coherent experience of agency and ownership. Among the experimental paradigms designed to probe the plasticity of the bodily self, the rubber hand illusion (RHI) has become one of the most influential. In the RHI, when a participant’s hidden real hand and a visible artificial hand are stroked synchronously, the participant often develops the compelling feeling that the artificial hand belongs to their own body. This illusion has been widely employed to study the mechanisms of multisensory integration and body ownership (Botvinick and Cohen 1998, Tsakiris and Haggard 2005). Traditional studies have quantified the illusion through proprioceptive drift (the perceived shift of the real hand’s position toward the rubber hand) and questionnaire ratings, while neuroimaging and electrophysiological studies have identified activity in premotor, parietal, and insular cortices as correlates of illusory ownership (Ehrsson et al. 2004, Tsakiris 2010).

Despite its established status, the RHI is not a binary “all-or-nothing” phenomenon. Instead, it represents a dynamic experiential process that unfolds gradually over time and varies across individuals. Phenomenological investigations have demonstrated that the illusion comprises multiple subjective state transitions. For instance, Valenzuela-Moguillansky et al. (2013) showed that participants’ first-person reports reveal a temporal microgenesis (i.e. the fine-grained temporal unfolding): beginning with an ambiguous sense of the rubber and real hands co-existing, moving through intermediate stages of partial incorporation of the artificial hand, and eventually leading to a more complete sense of ownership. These reports also highlight that the illusion involves not only the incorporation of the artificial hand but also changes in the felt presence of the real hand, including moments of disownership or altered tactile perception. Such findings challenge the traditional approach of collapsing subjective variability into averaged behavioral or neural responses, and instead call for a methodology that more precisely addresses individual differences in the processes of embodiment.

Previous studies have shown that the RHI has measurable temporal characteristics, including onset latency, gradual strengthening over time, and persistence or attenuation after stimulation (e.g. Kalckert and Ehrsson 2017, Perepelkina et al. 2018, Finotti et al. 2023). These findings suggest that the illusion develops over time, but they do not by themselves specify how the subjective structure of the experience changes during that process. Phenomenological studies have further suggested that the RHI may involve nuanced and evolving experiential changes rather than a single homogeneous state (Valenzuela-Moguillansky et al. 2013). Building on this literature, the present study examines whether the temporal unfolding of the RHI can be described in terms of distinct subjective states and how such states relate to electroencephalographic (EEG) network dynamics.

This challenge resonates with the framework of neurophenomenology, introduced by Varela (1996), which proposes to integrate first-person descriptions with third-person neurophysiological data through the principle of mutual constraints. Rather than discarding subjective reports as confounding factors, neurophenomenology treats them as indispensable data for understanding consciousness. Lutz et al. (2002) demonstrated the viability of this approach by clustering trials according to participants’ phenomenological context and showing distinct EEG synchrony patterns across these clusters. Their study revealed that neural variability, traditionally treated as a mere artifact, in fact reflects structured differences in subjective experience. More recently, neurophenomenological methods have been applied to the study of meditation and self-boundary dissolution, showing that subjective states such as the attenuation of self–world boundaries are graded, flexible, and accompanied by distinctive neural dynamics (Dor-Ziderman et al. 2016, Berkovich-Ohana et al. 2020, Lutz et al. 2025). These studies underscore the methodological value of systematically linking first-person data with neural measures to capture the dynamic and plastic nature of self-consciousness.

In the context of the RHI, adopting a neurophenomenological perspective allows us to investigate not only whether participants experience ownership of the rubber hand but also how this experience evolves through successive subjective states. Capturing these transitions requires methods capable of representing the fine-grained temporal unfolding of experience and linking it to dynamic neural processes. Our study advances this line of inquiry in three specific ways.

First, we focus on the temporal unfolding of subjective state transitions during the RHI. Rather than treating the illusion as a simple binary phenomenon, we adopt phenomenological interview methods (elicitation interview; Valenzuela-Moguillansky et al. 2013) to identify intermediate states, such as the simultaneous awareness of both the real and rubber hands or the partial transfer of tactile sensations. These microgenetic stages of the illusion reveal that body ownership is not established instantaneously but emerges gradually through dynamic reorganization of sensory and cognitive processes. Recognizing this unfolding process enables a more nuanced understanding of the embodied self.

Second, graph neural networks (GNNs) are introduced as an analytic tool for EEG functional connectivity data. While previous EEG studies of the RHI have primarily examined oscillatory power (Evans and Blanke 2013, Kanayama et al. 2021, Sciortino and Kayser 2022), event-related potentials (Rao and Kayser 2017, Zeller et al. 2015) or pairwise coherence (Zeller et al. 2016, Kanayama et al. 2017), such measures are limited in their ability to capture the large-scale organization of neural activity. GNNs provide a powerful framework for analyzing brain connectivity as a network, directly modeling the relational structure among electrodes (Ktena et al. 2018, Chang et al. 2021). By applying GNN-based clustering, distinct EEG network states can be identified that correspond to the phenomenologically identified stages of the illusion. This approach constitutes a methodological innovation, as it enables a data-driven discovery of neural clusters aligned with evolving subjective states, going beyond the traditional dichotomy of synchronous versus asynchronous stimulation.

Third, to establish correspondences between subjective state transitions and EEG network states, we employ Gromov–Wasserstein (GW) optimal transport. This method enables the alignment of two relational structures—even when they differ in dimensionality or metrics—by minimizing the distortion of their internal geometries (Villani 2009, Mémoli 2011, Peyré and Cuturi 2019, Zhang et al. 2023). In our case, the transition structure of subjective states (derived from phenomenological interview-based clustering) and the transition structure of EEG network states (obtained via GNN analysis) can each be treated as metric spaces. GW optimal transport provides an optimal mapping between them, thereby quantifying the structural correspondence between subjective and neural dynamics. This approach represents a novel computational formalization of the mutual constraints principle at the heart of neurophenomenology, bridging the subjective and neural domains in a systematic way.

By integrating these elements, we seek to transform variability—long regarded as confounding factors—into meaningful data that illuminate the dynamic constitution of the embodied self. In doing so, we reconceptualize the RHI not as a simple binary illusion but as a window into the ongoing process by which selfhood is enacted and reconfigured. More broadly, this study contributes to the advancement of neurophenomenology as a methodological framework, demonstrating how contemporary computational tools can operationalize the mutual constraints between first-person experience and third-person measures, thereby enriching the scientific study of consciousness. More specifically, the present study asks whether bodily selfhood is better understood not as a fixed or binary outcome, but as a dynamic process that emerges through intermediate experiential and neural transitions. From this perspective, the RHI serves not only as an experimental illusion paradigm, but also as a model system for investigating how the embodied self is gradually constituted, destabilized, and re-stabilized over time. The specific contribution of the present study is to examine whether this temporal constitution of bodily selfhood can be empirically captured by aligning first-person experiential state transitions with EEG-derived network-state transitions.

## Methods and materials

### Participants

Sixteen individuals participated in the experiment (eight men and eight women aged 21.25 ± 0.56). Participants were recruited from undergraduate and graduate students and reported no known neurological or psychiatric conditions. No apparent abnormalities of the upper limbs were reported. Handedness was assessed by self-report. Fifteen participants were right-handed and one was left-handed. The study procedures were approved by the ethics committee of Meiji University (24-574), and the study was conducted in accordance with the principles and guidelines of the Declaration of Helsinki. We obtained written informed consent from all participants. All participants were paid for their participation.

### Experimental design & procedure

**Figure 1:**
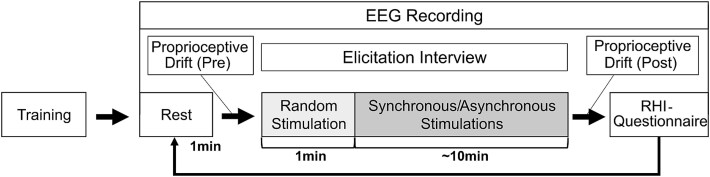
Schematic representation of the experimental procedure.

The overall experimental procedure is illustrated in Fig. 1. Participants sat at a table with their right hand placed behind a cardboard partition, so that only the rubber hand—positioned 15 cm to the right—was visible during induction. A gender-matched rubber hand was presented to each participant. Proprioceptive drift, measured as a behavioral index of the RHI, was assessed immediately before and after induction by asking participants to mark with a sticker the perceived location of their right hand; the experimenter then measured its distance from the actual hand. The RHI induction lasted for ~10 min, during which both the phenomenological interview and EEG recording (see below for details) were conducted.

We implemented two experimental conditions: the synchronous (Sync) condition and the asynchronous (Async) condition. In the Sync condition, two paintbrushes moved in unison, simultaneously stimulating the participant’s real right middle finger and the rubber hand. In the Async condition, the brushes moved with a temporal lag of more than 0.5 s, stimulating the same finger of the real hand and the rubber hand out of phase. Each participant completed both conditions, and the order of conditions was counterbalanced across participants.

To prevent participants from entering the illusory state at the very beginning of the induction, a 1-min random stimulation was applied before each condition, during which the experimenter stroked different spots on the real and rubber hands at different times. After this random stimulation, the experimenter instructed participants to begin reporting their moment-to-moment experiences. After each condition, participants completed the Japanese version of the RHI questionnaire (Botvinick and Cohen 1998, Shimada et al. 2009, 2014), using a Likert scale ranging from −3 to +3.

### Interview technique

We conducted the interviews using the elicitation interview approach (Vermersch 1999, Petitmengin 2006, Valenzuela-Moguillansky et al. 2013). This method guides participants to provide precise accounts of their experiences by helping them recall and articulate details through targeted prompts and questions (Petitmengin and Lachaux 2013), and it has been further developed into analytic procedures that enable systematic and intersubjectively validated study of first-person experience (Valenzuela-Moguillansky and Vásquez-Rosati 2019). The technique has revealed subtle perceptual changes in the RHI (Valenzuela-Moguillansky et al. 2013) and has also been applied to other domains, such as investigating how people experience and interpret data visualizations (Hogan et al. 2016). Together, these findings demonstrate that the elicitation interview is a powerful and versatile tool for eliciting fine-grained descriptions of subjective experience.

During the RHI induction, participants’ descriptions were collected in real time. They were encouraged to report their experiences spontaneously, especially when they noticed changes. The experimenter followed up on these reports with questions designed to elicit more detailed accounts, directing participants’ attention to the lived qualities of their sensations and prompting them to describe what the sensations were like and how they arose. For example, if a participant said, “It feels as if my hand is floating,” the experimenter might ask, “What is it like to feel that your hand is floating?” If participants remained silent for a while, they were gently prompted with open-ended questions such as, “How are you feeling now?” or “Do you feel anything at this moment?” With participants’ consent, all audio data were recorded during the RHI induction using a voice recorder (ICD-UX570FB, SONY, Japan). To align the EEG recording with the experiential descriptions, the onset of the IC recorder and EEG recording was manually synchronized at the beginning of the session. Thereafter, the timing of participants’ utterances was used to retrospectively relate the identified experiential states to the EEG timeline. To familiarize participants with the elicitation interview technique, we conducted a brief training session (<10 min) using riddles before the main experiment (see Supplementary Information for details).

### Interview data analysis

The audio recordings were transcribed by the experimenter, and the finalized transcripts were annotated with time stamps for each spoken segment. These transcripts then served as the basis for analysis. Before analysis, the data were preprocessed by removing responses from the random stimulation phase, excluding irrelevant utterances from the task phase, and reassembling fragmented descriptions into coherent statements. Irrelevant utterances were defined as those not directly related to participants’ experiential reports, such as requests for clarification (e.g. asking to repeat the question) and task-irrelevant remarks (e.g. indicating an upcoming cough).

Drawing on the analytical framework of Valenzuela-Moguillansky et al. (2013), we examined both the diachronic and synchronic structures of participants’ experiences. First, each participant’s interview was analyzed diachronically in order to reconstruct the temporal unfolding of subjective experience within each condition. This analysis focused on identifying transitions, fluctuations, and changes in bodily self-experience over time while preserving the original temporal order of participants’ experiential descriptions. Subsequently, a synchronic analysis was conducted across participants to identify recurrent experiential structures that appeared across different temporal contexts and experimental conditions.

Experiential categories were constructed using a combined ascending (data-driven) and descending (theory-informed) approach. The descending aspect involved classifying experiential descriptions according to phenomenological distinctions and previously proposed categories described by Valenzuela-Moguillansky et al. (2013). The ascending aspect involved identifying novel experiential patterns emerging from the present data and integrating similar descriptions across participants to form additional categories. Unlike the previous study (Valenzuela-Moguillansky et al. 2013), which analyzed the synchronous and asynchronous conditions separately, the present study aimed to identify phenomenological categories common to both conditions in order to examine subjective-state structures that could be systematically related to neural activity. At the same time, the temporal development of subjective experience was preserved throughout the analysis by maintaining the temporal order of participants’ experiential descriptions during segmentation and categorization. The final categorization was refined through iterative discussions among the authors, with particular emphasis on conceptual clarity and consistency across participants.

Building on this categorization, we constructed a subjective state transition model, based on the comparison of the individuals’ diachronic structures (see Supplementary Fig. 1), to capture how participants’ experiences unfolded over time. Each participant’s statements were first independently classified by two experimenters and then assigned to specific states through consensus. Transition probabilities were calculated by dividing the number of transitions to each destination state by the total number of transitions from a given state. Repeating this procedure for all states yielded the subjective state transition model, which is illustrated in the transition diagrams presented in the Results section.

### Electroencephalographic recording

EEG signals were recorded using a 24-bit bio-signal amplification unit (g.USBamp, g.tec Medical Engineering GmbH, Austria). Subjects were instructed to refrain from moving their eyes or bodies during recording. The signals were recorded with active Ag/AgCl electrodes. Electrical activity was amplified and digitized at 512 Hz with a band-pass filter of 0.5–100 Hz. Electrodes were mounted in an elastic cap and located at 30 positions according to the extended 10–20 system (Fp1, Fp2, F7, F3, Fz, F4, F8, FT7, FC3, FCz, FC4, FT8, T7, C3, Cz, C4, T8, TP7, CP3, CPz, CP4, TP8, P7, P3, Pz, P4, P8, POz, O1, O2). An active Ag/AgCl electrode was placed at the right ear lobe as a reference electrode and a passive Ag/AgCl electrode was placed at AFz as a ground electrode. The electrode positions of each subject were recorded using a 3D magnetic space digitizer (FASTRAK, Polhemus, Vermont, USA) to enable source-level analysis via standardized low-resolution brain electromagnetic tomography (sLORETA) software (Pascual-Marqui 2002).

### Electroencephalographic data analysis

**Figure 2:**
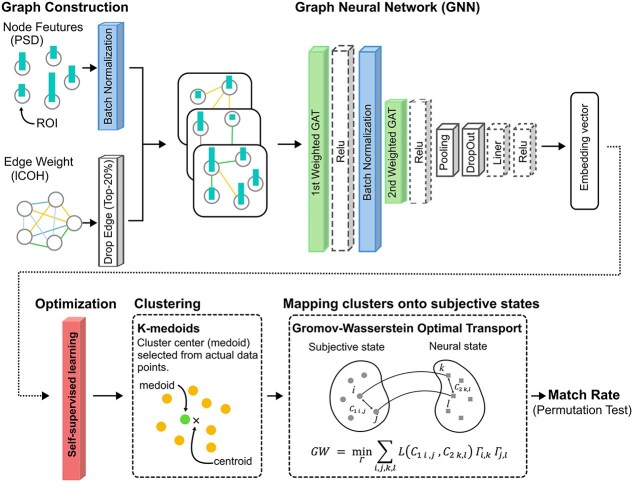
Schematic representation of the EEG analysis pipeline. Graphs were constructed with PSD-derived node features and lagged coherence (ICOH)-based edge weights (top 20%), processed using a weighted GAT-based GNN within a self-supervised learning framework, and subsequently clustered with K-medoids. The resulting clusters were mapped onto subjective states via GW optimal transport, and the correspondence was evaluated using a permutation test.

The overall EEG analysis workflow is illustrated in Fig. 2. First, spectral features were extracted from each region of interest (ROI), and functional connectivity was estimated based on lagged coherence. Next, these connectivity matrices were processed through a WeightedGAT-based GNN, which learned low-dimensional representations of brain network organization. The resulting embeddings were then clustered to identify distinct neural network states. Finally, the clustered neural states were aligned with the phenomenologically defined subjective states using GW optimal transport, providing a principled mapping between neural and subjective domains.

#### Preprocessing

The analysis was performed using EEGLAB version 2023.0 (Delorme and Makeig 2004) running on MATLAB 2023b (Mathworks, Natick, MA, USA). To attenuate power line noise, the CleanLine algorithm (Mullen 2012) was applied to all channels, targeting 50 and 100 Hz with a 2 Hz bandwidth and a 4 s sliding window (step size: 4 s; *P* < .05). A band-pass filter (1–60 Hz) was applied using a zero-phase Hamming windowed sinc FIR filter (order: 1690). Independent component analysis (ICA) was then performed using the extended Infomax algorithm (Makeig et al. 1996). Artifact-related components were identified using ICLabel (Pion-Tonachini et al. 2019). Components with a Brain probability of <30% were excluded, and components with an Eye probability >90% were additionally confirmed by visual inspection of the component maps before removal. On average, 6.3 ± 1.1 ICA components per participant were removed (range = 4–9). No separate interpolation of noisy electrodes or rejection of noisy data segments was performed. For topographic visualization and artifact rejection, electrode positions were transformed into a standard head model using sLORETA. ICA components associated with non-neural artifacts were identified and excluded.

#### ROI-definition

We defined 24 anatomically based ROIs (12 per hemisphere) informed by neuroanatomical knowledge of body ownership and multisensory integration (see Table 1). These included key regions such as the premotor cortex, parietal areas, and the insula. Each ROI was defined anatomically in source space using Brodmann area parcellation as implemented in the sLORETA framework. Further details on ROI selection are provided in the Supplementary Information. This set of ROIs served as the basis for extracting localized spectral features and computing pairwise functional connectivity.

**Table 1:** Regions of interest (ROIs) used for EEG analysis.

No.	ROI	Centroid			BA
		*X*	*Y*	*Z*	
1	L-PMv	−51.22807	15	13.1579	44, 45
2	L-PMd	−30.44964	3.291367	51.27698	6^*^, 8^*^
3	L-SPL	−20.25	−60.08333	52.63889	5 s, 7
4	L-IPL	−48.30827	−49.64286	35.50752	39, 40
5	L-SMC	−40.55263	−25.65789	50.65789	1^*^, 2^*^, 3^*^, 4^*^, 5
6	L-VC	−22.23433	−81.03542	5.163488	17, 18, 19
7	L-Ins	−38.92	−7.8	8.52	13
8	L-PCC	−10.57895	−48.42105	30.68421	23, 31
9	L-STG	−56.63213	−21.1658	−6.03627	21, 22
10	L-mPFC	−8.333333	16.22449	23.09524	24, 25, 32
11	L-MTL	−20.19608	−9.705882	−21.37255	28, 34, 35, 36
12	L-dlPFC	−30.58824	40.10504	20.73529	9, 10, 46
13	R-PMv	52.7193	15	13.33333	44, 45
14	R-PMd	31.20861	4.354305	51.19205	6^*^, 8^*^
15	R-SPL	21.03825	−60.30054	52.62295	5, 7
16	R-IPL	49.1875	−48.60417	36.35417	39, 40
17	R-SMC	42.10106	−25.66489	50.53191	1^*^, 2^*^, 3^*^, 4^*^, 5
18	R-VC	20.43702	−81.52956	5.051414	17, 18, 19
19	R-Ins	40.0813	−7.479675	9.308943	13
20	R-PCC	7.450331	−46.55629	30.62914	23, 31
21	R-STG	57.10648	−19.14352	−6.990741	21, 22
22	R-mPFC	6.829268	14.87805	22.2439	24, 25, 32
23	R-MTL	20.5	−10.2	−21.3	28, 34, 35, 36
24	R-dlPFC	31.56863	40	21.29412	9, 10, 46

^*^Includes only the lateral portion of the indicated Brodmann area.

#### Spectral feature extraction via source localization

Following preprocessing, EEG signals were segmented into consecutive, non-overlapping 10-s epochs. Segments shorter than 10 s at the end of each recording were discarded. For each epoch, power spectral density (PSD) was estimated using the fast Fourier transform with a Hann window, as implemented in the sLORETA software, to reduce spectral leakage. These PSD values were then averaged within standard frequency bands: delta (1–3 Hz), theta (4–7 Hz), alpha (8–13 Hz), beta1 (14–18 Hz), beta2 (19–22 Hz), beta3 (23–30 Hz), and gamma (31–45 Hz). Importantly, PSD estimation was not a primary analytic endpoint but served to generate features for subsequent source reconstruction and graph-based modeling. Specifically, the PSD features were projected into source space using sLORETA, which maps scalp EEG activity to cortical sources via a standardized three-shell spherical head model (6239 voxels at 5-mm resolution). sLORETA computed standardized current source density for each frequency band through regularized minimum norm solutions, yielding spatially resolved maps of neural oscillatory activity with correction for depth bias. For each ROI, amplitude features were extracted by averaging voxel-level current source density values within each anatomical region. These amplitude features served as node attributes in the GNN models, representing localized oscillatory activity at the network level.

#### Functional connectivity via lagged coherence

To capture physiologically meaningful functional coupling between ROIs while minimizing spurious effects of volume conduction, we computed lagged coherence using sLORETA’s connectivity analysis framework. This measure emphasizes phase-synchronous interactions with non-zero temporal delays, thereby reducing instantaneous correlations that often reflect common sources rather than genuine neural communication (Pascual-Marqui 2007). Lagged coherence was calculated for each frequency band and averaged across epochs to yield stable estimates of connectivity strength. The resulting connectivity matrices were subsequently used as input features for network-level modeling with GNNs.

#### WeightedGAT architecture and unsupervised representation learning

A GNN architecture termed weighted graph attention network (WeightedGAT) was implemented to learn informative representations from functional brain networks. For each frequency band, we constructed an undirected weighted graph in which nodes corresponded to ROIs (node features: spectral amplitude) and edges encoded inter-regional lagged coherence. To standardize feature scales and stabilize the attention mechanism, input node features were batch-normalized. In addition, to reduce spurious or physiologically implausible links, each connectivity matrix was thresholded to retain the top 20% of edges with the highest magnitude (Achard and Bullmore 2007, Bassett et al. 2008, Van Den Heuvel et al. 2008, Garrison et al. 2015, Jalili 2016), following evidence that fully connected graphs obscure meaningful structure and bias network metrics (Alexander-Bloch et al. 2010, Van Wijk et al. 2010, Friston 2011, Van Den Heuvel and Fornito 2014).

WeightedGAT comprised two successive multi-head graph attention layers, each computing attention-weighted neighborhood aggregations that adaptively modulated contributions based on topology and feature similarity. Batch normalization was applied between layers to stabilize training, and node-level outputs were pooled into a global graph embedding using an attention-based pooling module with learnable gates. The pooled vector was linearly projected into a low-dimensional embedding space for contrastive learning.

The model was trained in an unsupervised contrastive framework, where embeddings of each graph were normalized and compared within the batch. Positive similarity was defined by the diagonal of the similarity matrix (self-similarity), while off-diagonal entries contributed indirectly to the InfoNCE-style loss. A learnable temperature parameter was included, with L1 and L2 regularization applied to promote sparsity and constrain embedding norms. Optimization was performed using AdamW with an initial learning rate of 1e-5, cosine-annealing scheduling with warm restarts, and a dropout rate of 0.4. Training proceeded for up to 300 epochs with early stopping (patience = 100) based on validation loss stability.

All models were implemented in PyTorch and PyTorch Geometric. To ensure reproducibility, random seeds were fixed across all stochastic components, including data loaders, initialization routines, and model layers.

#### Cluster assignment and matching via Gromov–Wasserstein optimal transport

Following the extraction of low-dimensional graph embeddings, clustering was performed using the K-medoids algorithm, with the number of clusters set to match the number of states identified in participants’ interviews. This procedure separated the EEG data into distinct EEG network states within the feature space, providing a data-driven characterization of brain activity patterns.

To assess the correspondence between the EEG network states obtained from clustering and the subjective states, we applied GW optimal transport (Mémoli 2011), which aligns two metric spaces on the basis of their intrinsic structural similarity. This method allowed us to derive a soft correspondence between the distributions defined over the two domains—EEG network states and subjective states—even though their sample spaces were not directly comparable.

The GW distance was defined as the solution to the following optimization problem:


$$GW=\underset{\varGamma }{\min}\sum_{i,j,k,l}L\left({C}_{1\ i,j},{C}_{2\ k,l}\right)\ {\varGamma}_{i,k}\ {\varGamma}_{j,l}$$

here, ${C}_1\in{\mathbb{R}}^{n\times n}$ and ${C}_2\in{\mathbb{R}}^{m\times m}$denoted the intra-domain cost (or dissimilarity) matrices for the EEG network state space and the subjective state space, respectively. The indices $i,\kern0.5em j\in \left\{1,\dots, n\right\}$ referred to pairs of EEG network states, while $k,\kern0.5em l\in \left\{1,\dots, m\right\}$ referred to pairs of subjective states. The function $L$ represented a loss function that quantified discrepancies between pairwise relationships; in this study, we adopted the squared ${L}^2$ norm. The transport plan $\varGamma \in{\mathbb{R}}^{n\times m}$ mapped EEG network states to subjective states and was subject to marginal constraints $\varGamma{1}_m=p$ and ${\varGamma}^T{1}_n=q$, where $p$ and $q$ denoted the probability distributions over EEG network states and subjective states, respectively. These probability distributions were estimated from the empirical occurrence frequencies of each state across all participants. Transition probabilities among EEG network states and among subjective states were also computed and represented as Markov transition matrices. Their inverse matrices were used as the intra-domain cost matrices ${C}_1$ and ${C}_2$, which encoding functional dissimilarities between states. Using these matrices and distributions, the optimal transport plan ${\varGamma}^{\ast }$ was computed by minimizing the GW distance. Each entry of ${\varGamma}^{\ast }$ reflected the strength of association between a given EEG network state and a given subjective state. The most closely associated subjective state for each EEG network state was identified as the one with the highest corresponding transport weight.

After computing the most probable subjective state associated with each EEG network state within each frequency band using GW optimal transport, we assessed the alignment between the predicted EEG network state sequence and the actual time series of subjective state transitions reported by participants. To evaluate whether this alignment exceeded chance levels, we calculated the match rate between the EEG network state and subjective state sequences, and tested its statistical significance using a permutation test with 1000 iterations. Statistical significance was determined at a threshold of *P* < .05.

#### Degree centrality

Among graph-theoretical metrics, we focused on normalized degree centrality (DC), which quantifies the extent to which each brain region is directly connected to all others and provides a standardized measure of local functional connectivity (Zuo et al. 2012, Zhang et al. 2020). ROIs with higher DC are regarded as network hubs that facilitate information integration and coordination across distributed systems. Prior studies have shown its sensitivity to network reorganization in both cognitive and clinical contexts, including schizophrenia and end-stage renal disease (Li et al. 2016, Zhang et al. 2025).

To examine network hub properties within the EEG network states identified by GW optimal transport, each EEG network state was first assigned to its corresponding subjective state. Functional brain graphs were then averaged across all samples belonging to each subjective state and sparsified by retaining the top 20% of edge weights to emphasize strong functional links. Normalized DC was computed for each ROI using NetworkX (version 3.4.2), yielding values between 0 and 1 to enable comparisons across graphs of different sizes. This approach allowed us to capture state-dependent reorganization of functional hubs and to identify ROIs that served central roles during distinct subjective states.

### Statistical analysis

#### Traditional rubber hand illusion indices

As traditional RHI measures, proprioceptive drift and ownership questionnaire scores were compared between the Sync and Async conditions. Normality was tested using the Shapiro–Wilk test, followed by paired t-tests or Wilcoxon signed-rank tests as appropriate. All analyses were conducted in R (version 4.3.2; R Core Team 2025).

#### Electroencephalographic analysis

To evaluate the robustness of the GW-based correspondence between subjective states and EEG network states, we repeated the mapping procedure 20 times. This approach was motivated by the fact that in unsupervised learning, single-run results can be biased due to hyperparameter sensitivity, random initialization, and potential instability in optimization (Tsitsulin et al. 2023).

Second, to characterize the distinctiveness of EEG network states, we calculated the mean GW distance between all state pairs obtained through these mappings and used the Wilcoxon signed-rank test (Bonferroni corrected) to identify the frequency bands showing the greatest divergence. In this framework, the GW distance served as an index of dissimilarity among EEG network states.

Finally, to test whether state-related network reorganization differed systematically across experimental conditions and frequency bands, we examined changes in DC using linear mixed-effects models (LMMs) with a random intercept for each participant. LMMs were fitted using the lme4 package (Bates et al. 2015), and significant three-way interactions among Band, Condition, and State were followed by *post hoc* comparisons using estimated marginal means (emmeans; Lenth 2016). All *post hoc P*-values were adjusted using the false discovery rate (FDR) procedure.

## Results

### Behavioral measures

**Figure 3:**
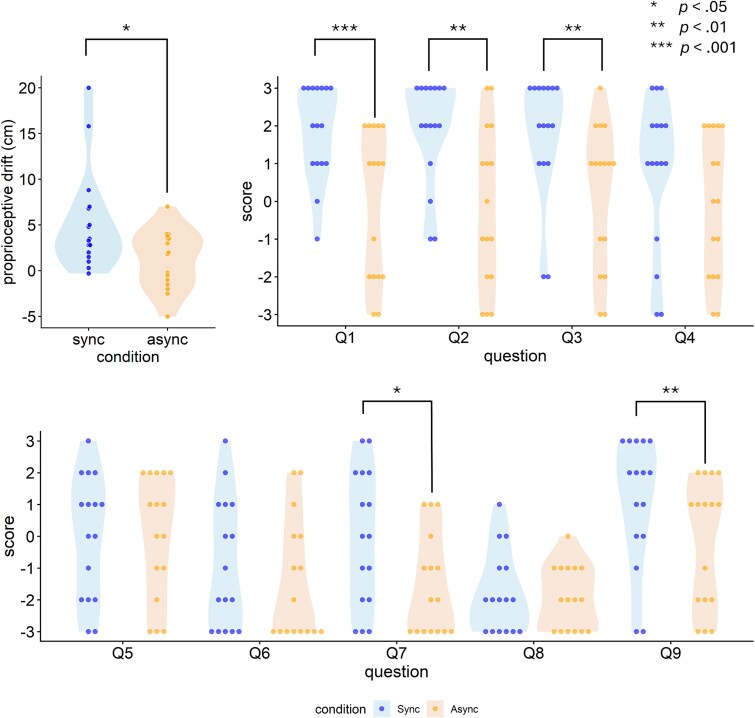
Proprioceptive drift and questionnaire results.

Figure 3 illustrates the results of the proprioceptive drift. Drift was significantly greater in the Sync condition than in the Async condition (*V* = 117, *P* = .009, *r* = 0.46), indicating that participants experienced a stronger illusion in the Sync condition. The questionnaire results further supported this finding. Significant differences were observed for items 1, 2, 3, 7, and 9 (Q1: *V* = 105, *P* < .001, *r* = 0.68; Q2: *V* = 108.5, *P* = .003, *r* = 0.53; Q3: *V* = 100.5, *P* = .001, *r* = 0.58; Q7: *V* = 56.5, *P* = .04, *r* = 0.36; Q9: *V* = 86, *P* = .002, *r* = 0.54; Fig. 3), with higher scores in the Sync condition than in the Async condition. Together, these results confirm that the synchronous versus asynchronous manipulation was effective and successfully reproduced the findings of previous studies.

### Interview

Analysis of the interview data revealed a range of experiences that shared common features across both the Sync and Async conditions. These experiences were grouped into subjective states, representing recurring phenomenological patterns independent of condition. Specifically, eight categories were identified, capturing both the diversity of participants’ subjective states and the distinct conceptual boundaries between them. Table 2 presents these categories together with representative experiential descriptions for each (see Supplementary Materials for detailed descriptions and additional examples).

**Table 2:** Identified subjective states and representative experiences.

State	Description	Example quotation
**State 1: Multisensory integration**		
**State 1–1: Perception of tactile stimulation**	Sensation of brush strokes applied to the real hand.	“It’s like being stroked with the tip of a brush.” (Sub12-Sync)
**State 1–2: Visuo-tactile congruence**	Awareness that visual and tactile inputs occur simultaneously.	“It feels like the model hand is being touched at the same timing and in the same spot as my real hand.” (Sub06-Sync)
**State 1–2**′**: Visuo-tactile incongruence**	Awareness of temporal mismatch between seen and felt touches.	“The timing of the model hand being stroked and my own sensation is different, so I can tell the timing is off.” (Sub10-Async)
**State 1–3: Perceptive dissonance**	Subtle sense of strangeness, surprise, or violation of expectation, without ownership.	“It’s like I brace myself for a moment each time the brush is about to touch me—I’m thinking, ‘Here it comes,’ but then it doesn’t, so I get this kind of restless, tingly feeling.” (Sub03-Async)
**State 1–4: Comparative attention**	Comparisons across modalities or situations to interpret sensations.	“If I focus on the model hand, it kind of looks like mine, but when I shift to my real hand, the location feels off.” (Sub10-Sync)
**State 1–5: Beginning of the incorporation of the RH**	Initial and tentative sense of ownership over the rubber hand.	“It feels like my own right hand inside the box is being shown as an image on the screen through the model’s hand.” (Sub06-Sync)
**State 2: Illusion**	Full experience of illusory ownership of the rubber hand.	“The hand in front of me feels like it’s my own.” (Sub01-Sync)
**State 2**′**: Absence of illusion**	No perceived change in hand ownership or spatial location.	“I can’t really feel like the fake hand is my own.” (Sub04-Async)

As in the framework of Valenzuela-Moguillansky et al. (2013), participants’ reports were organized into two overarching states: State 1 (Multisensory Integration), comprising five substates, and State 2/State 2′, corresponding to the illusory and non-illusory outcomes. Overall, the structure of subjective states was consistent with Valenzuela–Moguillansky’s model, although some substates exhibited differences in detail.

Within State 1, reports could be grouped into several distinct experiential categories. These included simple tactile sensations (State 1–1: Perception of Tactile Stimulation), as well as perceptions of congruence or incongruence between visual and tactile inputs (State 1–2: Visuo-tactile Congruence; State 1–2′: Visuo-tactile Incongruence). Some reports also involved a subtle sense of discomfort or strangeness (State 1–3: Perceptive Dissonance). In addition, a subset of reports was characterized by shifts of attention between sensory modalities or body locations during ongoing bodily experience (State 1–4: Comparative Attention). State 1–4 was characterized by statements in which participants explicitly directed attention to specific aspects of the situation and engaged in comparisons across sensory modalities or body locations (e.g. “If I focus on the model hand…”). In contrast, State 1–3 primarily reflected perceptual responses to sensory stimulation, including feelings of mismatch or ambiguity arising from visuo-tactile discrepancies. A large proportion of participants also reported an emerging sense of ownership of the rubber hand (State 1–5: Beginning of the Incorporation of the RH).

State 2 represents the illusory experience, characterized by greater confidence and more pronounced features compared to State 1–5. Within this state, we identified four types of experiences: (i) Typical RHI, involving a sense of ownership toward the rubber hand, which may be accompanied by a sense of delocalization and/or misplacement of the real hand, and/or a sense of agency toward the rubber hand (e.g. “It feels like my hand has come in front of me, into a position where I can see it”, Sub05-Sync); (ii) Double Hand Illusion (DHI), in which participants felt ownership of both the rubber hand and their own real hand simultaneously (e.g. “Somehow it feels like I have two hands”, Sub02-Sync); (iii) Phantom Tactile Sensation (PTS), where a tactile sensation was perceived in the real hand even when only the rubber hand was touched, occurring exclusively in the Async condition (e.g. “It felt as if my real hand was being stroked at the same time, even though only the fake hand was touched”, Sub07-Async); and (iv) Continuous Weak Illusion (CWI), in which the weak ownership feelings of State 1–5 persisted for more than 3 min, indicating a stabilized weak form of the illusion. These forms of illusory experience are consistent with those described in previous studies, such as Valenzuela-Moguillansky et al. (2013).

State 2′ represents the absence of illusion, typically observed in the latter part of the induction period. Descriptions within this state indicated no change in the perceived location of the real hand and no sense of ownership toward the rubber hand.

Table 3 shows the number of participants who experienced any illusion and each type of illusion. The number of participants classified as experiencing the illusion was derived primarily from the phenomenological interview data rather than directly from questionnaire thresholds. Following the standard definition of the RHI, reports reflecting ownership of the rubber hand and tactile referral were treated as core indicators of the illusion, together with closely related experiential variations. Accordingly, 12 out of 16 participants experienced the illusion in the Sync condition and 7 out of 16 in the Async condition. Thus, most participants experienced the illusion in the Sync condition, whereas fewer than half did so in the Async condition. Although it is surprising that such a high proportion of participants reported the illusion under the Async condition, this tendency is consistent with previous findings. In the study by Valenzuela-Moguillansky et al. (2013), 7 out of 10 participants also reported illusory experiences under asynchronous stimulation. These findings from the interview data are therefore in line with earlier reports showing that the illusion is more readily induced under synchronous stimulation, but can nevertheless occur under asynchronous conditions.

**Table 3:** Illusion types and participant numbers by condition.

	Sync	Async
The number of participants experienced the illusion	12	7
Rubber Hand Illusion (RHI)	8	2
Double Hand Illusion (DHI)	1	1
Phantom Tactile Sensation (PTS)	0	4
Continuous Weak Illusion (CWI)	3	0

To examine the relationship between interview-based classifications and questionnaire measures, we compared these classifications with participants’ ratings on the RHI questionnaire. Overall, a high degree of correspondence was observed. Most participants classified as experiencing the illusion (10/12 in the synchronous condition and 5/7 in the asynchronous condition) gave positive ratings (≥ 1) on the classical illusion-related items (Q1–Q3), with the ownership-related item Q3 endorsed by all participants classified as experiencing the illusion. In addition, participants reporting the double-hand illusion generally gave positive ratings on Q5, which corresponds to the experience of having more than one hand. In contrast, participants who did not reach higher-level experiential states generally gave negative ratings (≤ −1) on the core illusion-related items, with only one participant showing weakly positive responses.

### Subjective state transition model

**Figure 4:**
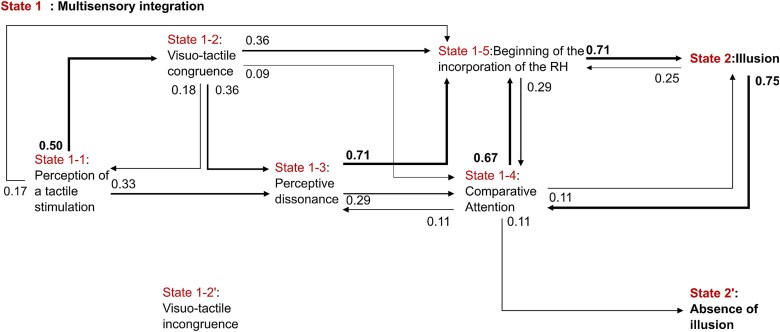
Subjective state transition map in the sync condition.

**Figure 5:**
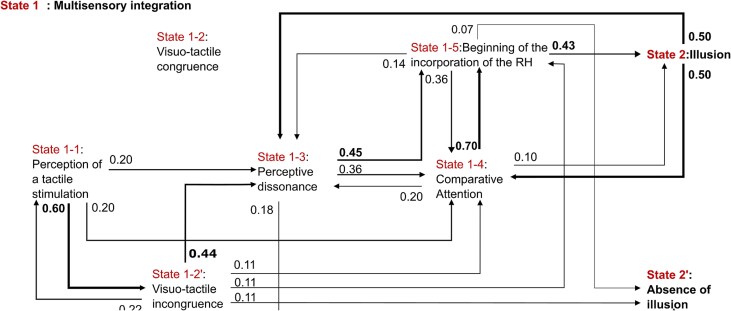
Subjective state transition map in the Async condition.

Figures 4 and 5 illustrate the transition probabilities between the eight subjective states, depicting the dynamic progression toward either the illusory or non-illusory state. The ordering of states was derived from qualitative comparisons of transition patterns across participants, supported by the frequency of observed transitions. This ordering reflects a typical progression rather than a fixed temporal sequence. Within this schematic arrangement, State 1–5 was positioned closer to the illusion state, whereas State 1–4 was relatively more distant.

Under the Sync condition, certain pathways were predominant, indicating smoother progression toward the illusion. Participants typically transitioned either directly from State 1–2 to State 1–5 (36%) or via State 1–3 before reaching State 1–5 (36%). State 1–4 most often appeared prior to the emergence of ownership-related experiences, but could also occur afterward, although such transitions were relatively rare. Once in State 1–5, the majority of participants progressed to the illusory state (71%), suggesting a relatively stable trajectory toward the RHI.

In contrast, transitions under the Async condition were more varied and unstable. From State 1–2′, transitions to State 1–3 occurred in 44% of cases, but transitions to several other higher states (excluding the illusory state) were also observed. Moreover, transitions from State 1–5 to the illusory state occurred in only 43% of cases, considerably lower than in the Sync condition. Participants frequently returned to earlier states (e.g. State 1–3 or State 1–4) or transitioned to non-illusory states, reflecting a disrupted and less stable progression.

Overall, these results demonstrate that the progression toward the RHI is smoother and more stable under synchronous stimulation, whereas in the asynchronous condition, the process is more complex, less stable, and often fails to culminate in a sustained illusory state.

### Electroencephalographic

#### Match rate

The interview analysis revealed eight subjective states, which were used to determine the number of clusters—i.e. the number of EEG network states—in the K-medoids algorithm. A mapping between these EEG network states and subjective states within each frequency band was then established using GW optimal transport. Across all frequency bands exclude beta3, significant match rates were obtained in at least 14 out of 20 runs (permutation test, *P* < .05; see Table 4).

**Table 4:** Match rates between EEG network states and subjective states obtained by GW optimal transport.

Frequency	Condition	Mean	SD	Max (*P*)	Significant runs out of 20 (*P* < .05, permutation test)
Delta	Sync	0.259	0.014	0.282 (*P* < .001)	17
	Async	0.227	0.017	0.254 (*P* < .001)	18
Theta	Sync	0.264	0.017	0.310 (*P* < .001)	18
	Async	0.227	0.025	0.266 (*P* < .001)	18
Alpha	Sync	0.264	0.021	0.304 (*P* < .001)	15
	Async	0.230	0.020	0.256 (*P* < .001)	18
Beta1	Sync	0.256	0.018	0.289 (*P* < .001)	14
	Async	0.219	0.025	0.276 (*P* < .001)	17
Beta2	Sync	0.267	0.016	0.299 (*P* < .001)	18
	Async	0.227	0.022	0.266 (*P* < .001)	16
Beta3	Sync	0.251	0.013	0.289 (*P* < .001)	11
	Async	0.219	0.026	0.283 (*P* < .001)	16
Gamma	Sync	0.256	0.018	0.292 (*P* < .001)	14
	Async	0.233	0.011	0.256 (*P* < .001)	20

Following the mapping process, no EEG network states were assigned to State 1–4. This may reflect the heterogeneous nature of this state, which encompasses multiple experiential aspects. As a result, the available data may have been insufficient to extract a consistent EEG pattern shared across instances of this state. Therefore, subsequent analyses focused on the remaining seven states, with State 1–4 excluded.

To identify the frequency bands with the strongest state divergence, we first computed the average functional connectivity matrix for each mapped state and then calculated the mean GW distance across all state pairs within each band. The mean GW distance served as an index of network dissimilarity. A Wilcoxon signed-rank test revealed that the theta and beta2 bands yielded the lowest mean GW distances, indicating the most pronounced divergence between states (full pairwise results are provided in the Supplementary Information; Supplementary Fig. 2 and Supplementary Table 1).

#### The mean degree centrality

**Figure 6:**
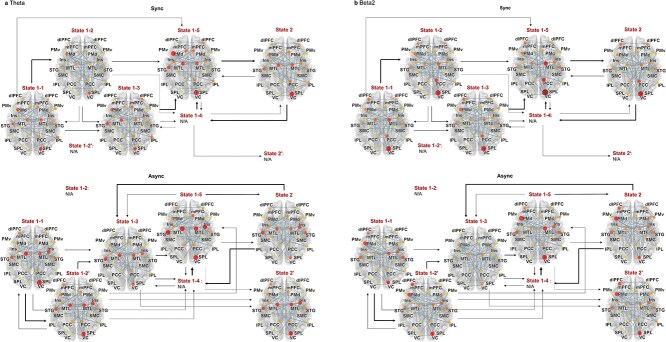
EEG network hub structure corresponding to each subjective state. Panels show neural states in the (a) Theta and (b) Beta2 bands for the Sync (top) and Async (bottom) conditions. Arrows represent transition probabilities between subjective states, with thicker arrows indicating higher probabilities. Lines within each brain map represent functional connectivity between ROIs, with only the top 20% strongest edges displayed. Circles denote the DC of each ROI; larger circle size and warmer colors indicate greater DC. N/A indicates that no EEG network was assigned to the corresponding subjective state.

To investigate how specific brain regions acted as network hubs across subjective states, we focused on DC, an index of how strongly each region is connected to the rest of the network. EEG network configurations for each subjective state in the Sync and Async conditions are shown in Fig. 6 for the theta and beta2 bands. The spatial distribution of high-DC regions indicated that distinct sets of ROIs served as network hubs depending on the subjective state and frequency band. To quantitatively assess these differences, we applied LMMs with a random intercept for each participant, examining ROI-specific effects of State, Condition, and Band. ROIs showing significant interactions were then analyzed further to identify how hub configurations varied across conditions and frequency bands.

**Figure 7:**
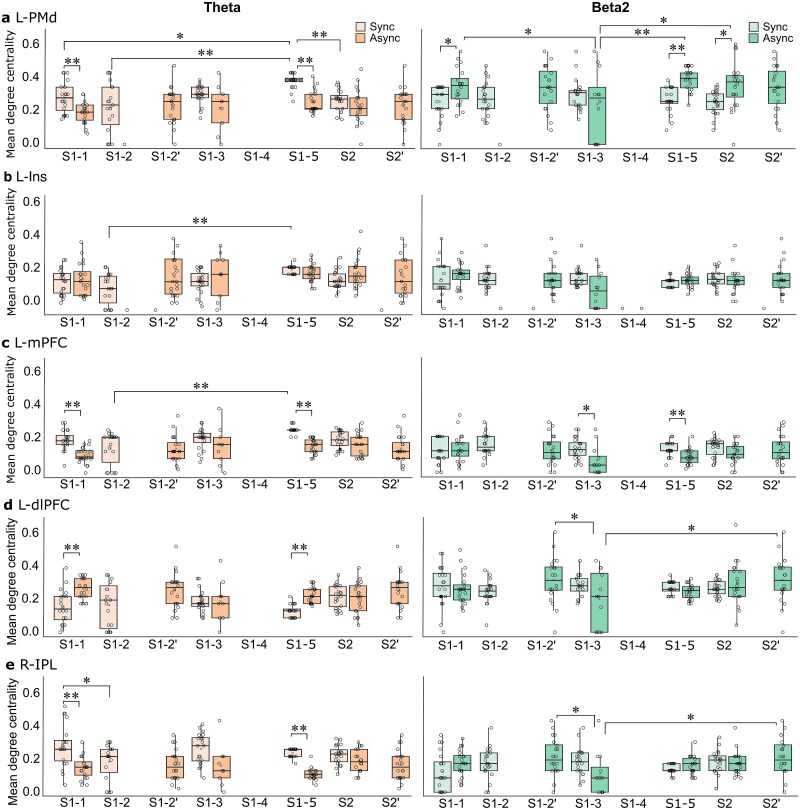
Mean DC in each ROI. Panels show results for each ROI: (a) L-PMd, (b) L-Ins, (c) L-mPFC, (d) L-dlPFC, and (e) R-IPL, with Theta-band results on the left and Beta2-band results on the right. Subjective states are abbreviated on the x-axis as S1–1 to S2´. Mean values are shown as crosses and individual data points as circles. N/A indicates states with no assigned EEG network. (*P < .05, **P < .01, FDR-corrected).

The LMMs revealed that significant three-way interactions among State, Condition, and Band in several ROIs: L-PMd (*F*(3, 420) = 3.61, *P* < .05, ${\eta}_p^2$ = 0.025), L-Ins (*F*(3, 402.09) = 3.12, *P* < .05, ${\eta}_p^2$ = 0.023), L-dlPFC (*F*(3, 402.47) = 3.33, *P* < .05, ${\eta}_p^2$ = 0.024), L-mPFC (*F*(3, 420) = 3.88, *P* < .01, ${\eta}_p^2$ = 0.027), and R-IPL (*F*(3, 401.72) = 3.96, *P* < .01, ${\eta}_p^2$ = 0.029). For these ROIs, *post hoc* comparisons using *emmeans* were conducted separately for each frequency band to examine State × Condition effects within bands. All reported *post hoc P*-values were FDR-corrected. The results for each frequency band are presented below (Fig. 7).

For clarity, the significant *post hoc* comparisons of normalized DC in the theta and beta2 bands are summarized in Table 5.

**Table 5:** Summary of significant *post hoc* comparisons of normalized DC in the theta and beta2 bands.

Band	ROI	Comparison	Estimate	*P*
**Theta**	**L-PMd**	Sync: State 1–5 > State 1–1	0.100	.035
		Sync: State 1–5 > State 1–2	0.159	<.01
		Sync: State 1–5 > State 2	0.120	<.01
		State 1–1: Sync > Async	0.087	<.01
		State 1–5: Sync > Async	0.129	<.01
	**L-Ins**	Sync: State 1–5 > State 1–1	0.115	<.01
	**L-mPFC**	Sync: State 1–5 > State 1–2	0.113	<.01
		State 1–1: Sync > Async	0.093	<.01
		State 1–5: Sync > Async	0.099	<.01
	**L-dlPFC**	State 1–1: Sync < Async	−0.111	<.01
		State 1–5: Sync < Async	−0.099	<.01
	**R-IPL**	Sync: State 1–1 > State 1–2	0.095	.025
		State 1–1: Sync > Async	0.130	<.01
		State 1–5: Sync > Async	0.130	<.01
**Beta2**	**L-PMd**	Async: State 1–3 < State 1–1	−0.108	.044
		Async: State 1–3 < State 1–5	−0.150	<.01
		Async: State 1–3 < State 2	−0.114	.031
		State 1–1: Async > Sync	0.091	.019
		State 1–5: Async > Sync	0.125	<.01
		State 2: Async > Sync	0.104	.018
	**L-mPFC**	State 1–3: Async < Sync	−0.073	.032
		State 1–5: Async < Sync	−0.046	<.01
	**L-dlPFC**	Async: State 1–3 < State 1–2′	−0.117	.026
		Async: State 1–3 < State 2′	−0.117	.026
	**R-IPL**	Async: State 1–3 < State 1–2′	−0.103	.025
		Async: State 1–3 < State 2′	−0.103	.025

***Theta band***: In the **L-PMd**, *post hoc* analyses revealed significant condition- and state-specific effects. Under the Sync condition, mean DC in State 1–5 was significantly higher than in State 1–1 (estimate = 0.10, *SE* = 0.033, *t*(422.09) = 3.01, *P* = .031), State 1–2 (estimate = 0.159, *SE* = 0.034, *t*(423) = 4.74, *P* < .01), and State 2 (estimate = 0.12, *SE* = 0.033, *t*(422.09) = 3.61, *P* < .01). Furthermore, mean DC was significantly higher in the Sync condition compared with the Async condition for State 1–1 (estimate = 0.087, *SE* = 0.026, *t*(21.05) = 3.38, *P* < .01) and State 1–5 (estimate = 0.129, *SE* = 0.016, *t*(21.05) = 8.04, *P* < .01).

In the **L-Ins**, *post hoc* analyses showed that in the Sync condition, mean DC in State 1–5 was significantly higher than in State 1–1 (estimate = 0.115, *SE* = 0.026, *t*(422.96) = 4.36, *P* < .01).

In the **L-mPFC**, significant condition- and state-specific effects were also found. Under the Sync condition, mean DC in State 1–5 was significantly higher than in State 1–2 (estimate = 0.113, *SE* = 0.022, *t*(423.12) = 5.08, *P* < .01). Additionally, Sync values were significantly higher than Async in both State 1–1 (estimate = 0.093, *SE* = 0.017, *t*(21.05) = 5.52, *P* < .01) and State 1–5 (estimate = 0.099, *SE* = 0.009, *t*(21.05) = 10.90, *P* < .01).

In the **L-dlPFC**, *post hoc* analyses revealed that mean DC was significantly lower in the Sync condition compared with the Async condition for State 1–1 (estimate = −0.111, *SE* = 0.025, *t*(21.05) = −4.35, *P* < .01) and State 1–5 (estimate = −0.099, *SE* = 0.014, *t*(21.05) = −7.07, *P* < .01).

In the **R-IPL**, *post hoc* analyses showed significant condition- and state-specific effects. Within the Sync condition, mean DC in State 1–1 was significantly higher than in State 1–2 (estimate = 0.095, *SE* = 0.028, *t*(423.09) = 3.37, *P* = .025). Moreover, mean DC was significantly higher in Sync than Async for both State 1–1 (estimate = 0.130, *SE* = 0.032, *t*(21.05) = 4.02, *P* < .01) and State 1–5 (estimate = 0.130, *SE* = 0.009, *t*(21.05) = 14.75, *P* < .01).

***Beta2 band***: In the **L-PMd**, *post hoc* analyses revealed significant condition- and state-specific effects. In the Async condition, mean DC in State 1–3 was significantly lower than in State 1–1 (estimate = −0.108, *SE* = 0.038, *t*(431.08) = −2.80, *P* = .044), State 1–5 (estimate = −0.150, *SE* = 0.038, *t*(431.08) = −3.91, *P* < .01), and State 2 (estimate = −0.114, *SE* = 0.038, *t*(431.08) = −2.97, *P* = .031). Mean DC was also significantly higher in the Async condition compared with the Sync condition for State 1–1 (estimate = 0.091, *SE* = 0.033, *t*(21.05) = 2.75, *P* = .019), State 1–5 (estimate = 0.125, *SE* = 0.018, *t*(21.05) = 6.93, *P* < .01), and State 2 (estimate = 0.104, *SE* = 0.036, *t*(21.05) = 2.87, *P* = .018).

In the **L-mPFC**, significant condition-specific effects were also found. Mean DC was significantly lower in the Async condition compared with the Sync condition in State 1–3 (estimate = −0.073, *SE* = 0.028, *t*(19.32) = −2.64, *P* = .032) and State 1–5 (estimate = −0.046, *SE* = 0.014, *t*(21.05) = −3.30, *P* < .01).

In the **L-dlPFC**, significant condition-specific effects were observed. In the Async condition, mean DC in State 1–3 was significantly lower than in State 1–2′ (estimate = −0.117, *SE* = 0.035, *t*(430.35) = −3.30, *P* = .026) and State 2′ (estimate = −0.117, *SE* = 0.035, *t*(430.35) = −3.30, *P* = .026).

In the **R-IPL**, *post hoc* analyses revealed significant state-specific effects. Mean DC in State 1–3 was significantly lower than in State 1–2′ (estimate = −0.103, *SE* = 0.032, *t*(430.83) = −3.20, *P* = .025) and State 2′ (estimate = −0.103, *SE* = 0.032, *t*(430.83) = −3.20, *P* = .025).

## Discussion

The present study combined phenomenological interviews with EEG network analyses to investigate the dynamic processes underlying the RHI. By integrating subjective reports with neural network states, we identified distinct experiential states and demonstrated their correspondence with EEG network dynamics across different frequency bands. This multimodal approach allowed us to capture not only the conditions under which the illusion is more likely to occur but also the fine-grained transitions leading toward or away from the illusory state. Importantly, the significance of these findings extends beyond the RHI itself. Rather than treating bodily selfhood as a fixed or binary property, the present results suggest that it may emerge through multiple intermediate experiential and neural transitions. More specifically, the study shows that the dynamic formation of bodily selfhood can be examined in terms of a structured correspondence between first-person experiential changes and large-scale neural network reorganization. In this sense, the study contributes not only to a more fine-grained understanding of the RHI, but also to an empirical framework for investigating the temporal constitution of the embodied self.

### Dynamics of subjective experience

The behavioral findings confirmed that the experimental manipulation was effective: proprioceptive drift and questionnaire scores were significantly greater in the synchronous condition than in the asynchronous condition. These results are consistent with the classical literature on the RHI (Botvinick and Cohen 1998, Tsakiris and Haggard 2005, Shimada et al. 2009, 2014, Suzuki et al. 2013) and subsequent replications showing synchrony between visual and tactile stimulation as a critical determinant of the illusion. We defined Q1–Q3 *a priori* as the primary illusion-related questionnaire items following previous work (Botvinick and Cohen 1998, Tosi et al. 2024), as these items are generally considered to reflect relatively robust aspects of the RHI, particularly tactile referral and ownership. At the same time, significant effects were also observed for Q7 and Q9, suggesting that synchronous stimulation may induce additional experiential dimensions beyond the classical ownership-related components of the illusion. Previous factor-analytic and network-based studies have likewise examined questionnaire items conceptually similar to Q7 and Q9 and reported associations with more central illusion-related measures, including ownership (Romano et al. 2021, Tosi et al. 2024). In addition, the use of elicitation interviews in the present study may have encouraged participants to attend more closely to both visual and bodily aspects of their experience, potentially enhancing experiential dimensions beyond the classical ownership components. These findings further support the view that the RHI is not a single homogeneous experience, but rather a multidimensional phenomenon involving partially distinct experiential components. Importantly, these behavioral indices not only validated the success of our paradigm but also provided a benchmark for interpreting the more fine-grained experiential and neural data.

Beyond these confirmations, the phenomenological interviews revealed that the RHI is not an all-or-none phenomenon but a dynamic process composed of multiple subjective states. Participants described a progression from basic tactile perception, through phases of visuo-tactile congruence or incongruence, to partial incorporation of the rubber hand, and ultimately to either an illusory or non-illusory outcome. The proportion of participants reporting illusion-related experiences in the present study (75%) is broadly consistent with previous findings suggesting that approximately two-thirds to three-quarters of participants experience the illusion under synchronous stimulation (e.g. Riemer et al. 2019). Similar proportions have also been reported in studies employing elicitation interviews (e.g. Valenzuela-Moguillansky et al. 2013). At the same time, a subset of participants did not transition into a stable illusion-related state. In contrast to many participants who experienced the illusion in the synchronous condition and transitioned relatively directly from State 1–5 to the illusion state, these participants tended to remain in intermediate experiential states, suggesting difficulty in resolving the perceptual uncertainty associated with Comparative Attention (State 1–4). Notably, even under asynchronous stimulation—where the illusion was expected to be weaker—nearly half of the participants reported some form of illusory experience, paralleling the findings of Valenzuela-Moguillansky et al. (2013). Such evidence suggests that ownership-related experiences can arise even when sensory congruence is disrupted, reflecting the resilience and variability of the underlying processes. At the same time, the illusion-related experiences observed under asynchronous stimulation were often phenomenologically distinct from the more stable ownership experiences observed under synchronous stimulation, frequently involving perceptual ambiguity, comparative attention, or less stable forms of bodily incorporation. One possible explanation is that the phenomenological interview itself encouraged participants to attend more carefully to subtle bodily sensations and experiential fluctuations that might otherwise remain unreported in standard questionnaire-based paradigms. In this respect, the elicitation interview may have increased sensitivity to weaker or atypical forms of embodiment-related experience. These findings should also be considered in relation to ongoing discussions concerning phenomenological control, suggestibility, and demand characteristics in the RHI literature (e.g. Lush et al. 2020, Lush and Seth 2022). Although the present study was not designed to directly assess such traits, their possible contribution cannot be excluded and should be addressed in future work.

The differentiation between substates also highlights how participants negotiate conflicting sensory signals. For example, perceptual dissonance (“something feels odd”) contrasts with comparative attention (active shifts of attention between seen and felt inputs), suggesting that the illusion involves multiple experiential dimensions related to both sensory integration and attentional modulation. The coexistence of such phenomenologically distinct experiential states highlights the importance of neurophenomenological approaches (Varela 1996, Depraz et al. 2003, Petitmengin 2006), which aim to systematically relate first-person descriptions with physiological measures. In this respect, the RHI provides a unique experimental window into how the embodied self emerges through the interplay of automatic perceptual mechanisms and reflective awareness (Gallagher and Zahavi 2008). By combining systematic experiential reports with behavioral and neural indices, neurophenomenological methods reveal layers of the illusion that remain invisible to traditional behavioral measures such as proprioceptive drift and questionnaire.

Although the present study identified several phenomenologically distinct illusion types, it did not include a formal subtype-specific EEG analysis because several illusion subtypes were represented by only a small number of cases and were unevenly distributed across conditions, making any statistical comparison between illusion subtype and EEG pattern underpowered and potentially misleading. Future studies with larger and more balanced samples will be needed to determine whether these different illusion subtypes are associated with distinguishable neural patterns. The present findings should be considered in relation to previous studies showing that the RHI has measurable temporal characteristics. Earlier work has shown, e.g. that the illusion has a measurable onset latency (Kalckert and Ehrsson 2017), may strengthen progressively during induction (Perepelkina et al. 2018, Finotti et al. 2023), and may persist or decay after stimulation (Perepelkina et al. 2018, Finotti et al. 2023). Prior work has also suggested that different aspects of the illusion may show dissociable temporal profiles, and that ownership-related experiences can be accompanied by changes such as disownership over time (Rohde et al. 2011, Lane et al. 2017). The contribution of the present study is not simply to restate that the RHI changes over time, but to suggest that this temporal evolution may be structured in terms of multiple distinguishable subjective states. In this sense, the present results extend the temporal-evolution literature by proposing that onset, strengthening, and attenuation may reflect not only quantitative changes in illusion strength, but also transitions among qualitatively distinct experiential states that may involve partially distinguishable EEG network dynamics (Valenzuela-Moguillansky et al. 2013).

### Correspondence between subjective states and electroencephalographic network states

A central contribution of this study is the demonstration of a systematic correspondence between phenomenologically defined subjective states and EEG-derived network states. Using GW optimal transport, clusters from functional connectivity patterns were aligned with the eight states identified through interviews, providing a principled framework for bridging lived experience and neural dynamics (Mémoli 2011, Peyré and Cuturi 2019). Significant match rates across multiple frequency bands indicate that subjective states are not arbitrary but reflected in reproducible neural configurations.

This finding supports the neurophenomenological claim that first-person data can constrain and enrich neuroscientific models (Varela 1996, Lutz and Thompson 2003). Rather than anecdotal, the temporal transitions reported in interviews corresponded to measurable neural reorganization. Notably, not all states were equally represented—State 1–4 did not yield a stable correspondence, likely due to sparse introspective reports—highlighting both the potential and the limitations of integrating phenomenological and neural data.

By mapping subjective and EEG network states, our results extend prior RHI research focused mainly on global contrasts between synchronous and asynchronous stimulation (e.g. Tsakiris and Haggard 2005, Ehrsson 2007). Instead, we show that the illusion unfolds through multiple intermediate states, each with distinct neural signatures, offering a richer account of how illusory experiences emerge and sometimes persist even under incongruent conditions.

### Frequency-specific network reorganization

Our results demonstrated that theta and beta2 bands exhibited the strongest divergence across subjective states, suggesting that these frequency ranges play a pivotal role in shaping the dynamics of the RHI. In the theta band, regions such as the L-PMd, L-Ins, L-mPFC, and R-IPL showed state- and condition-dependent changes in DC, indicating that theta oscillations contribute to multisensory integration and the emergence of body ownership. This interpretation aligns with prior findings that theta activity supports cross-modal binding and the detection of mismatches between sensory modalities (Tzovara et al. 2015, Senoussi et al. 2022). Moreover, theta oscillations have been linked to top–down attentional control and monitoring of internal versus external signals (Cavanagh and Frank 2014), processes that appear essential for negotiating perceptual dissonance and comparative attention during the illusion.

Firstly, in the theta band, the left PMd showed an increase in DC from State 1–1 and State 1–2 to State 1–5, followed by a decrease from State 1–5 to State 2 under the Sync condition. This suggests that the premotor cortex plays an active role during the transitional phase in which ownership over the rubber hand is emerging but not yet fully consolidated. Rather than supporting the maintenance of an already stabilized illusion, PMd activity appears to peak when participants are negotiating whether the rubber hand is incorporated as their own (State 1–5). Recent work using the full-body illusion paradigm has also emphasized the role of the premotor cortex in ownership experiences (Higo et al. 2025). In that study, the left premotor cortex remained significantly activated even when only visual stimuli were presented following synchronous visuo-tactile induction, thereby producing a visuo-tactile discrepancy. This activation was interpreted as reflecting the reconciliation of sensory conflicts to sustain the sense of ownership, a finding consistent with the present results. Taken together, these findings indicate that the premotor cortex contributes critically to the formation and negotiation of body ownership—particularly in intermediate, unstable states such as State 1–5—rather than simply maintaining an already established illusion.

In the left insula, an increase in DC from State 1–2 to State 1–5 in the theta band under the Sync condition likely reflects network-level processes that support the conscious emergence of body ownership. This interpretation is consistent with the insula’s role as a multimodal integration hub, combining interoceptive and exteroceptive signals while assigning emotional salience to these inputs (Simmons et al. 2013, Gogolla 2017, Zhang et al. 2024). Converging evidence from lesion studies further supports this view: damage to the left posterior insula disrupts self–other bodily distinctions and alters bodily self-awareness (Berlucchi and Aglioti 2010, Heydrich and Blanke 2013, Schaller et al. 2021). Connectivity between the insula and limbic structures such as the amygdala may further facilitate the integration of sensory and affective information, supporting the perception that an external object is incorporated into one’s own body (Kurth et al. 2010, Matuz-Budai et al. 2022, Crucianelli et al. 2024).

A similar increase was also observed in the left mPFC. The mPFC has been widely implicated in self-referential processing by integrating interoceptive and exteroceptive information (Northoff et al. 2006). Taken together, these findings suggest a division of labor: the insula provides the sensory–affective foundation of body ownership through multimodal integration, whereas the mPFC contributes higher-order transformations necessary for incorporating the rubber hand into the self-body representation. Thus, increases in DC within these regions indicate that both the insula and mPFC became more integrally embedded within large-scale networks during the emergence of body ownership, with the insula anchoring sensory–affective integration and the mPFC coordinating higher-order transformations. Importantly, this network-level interpretation aligns with participants’ subjective reports at State 1–5, which captured the initial emergence of ownership over the rubber hand.

In the right IPL, a decrease in DC from State 1–1 to State 1–2 in the theta band under the Sync condition likely reflects an early disruption of visuospatial body representations, marking the onset of illusion-related processes (Seghier 2013, Bréchet et al. 2018, Chancel et al. 2022). Across the left PMd, left mPFC, and right IPL, DC in States 1–1 and 1–5 was greater under Sync than Async conditions, suggesting stronger engagement of multisensory integration and ownership processes during synchronous stimulation (Gentile et al. 2011, Brozzoli et al. 2014, Ehrsson 2020). By contrast, the left dlPFC showed lower centrality under Sync than Async, consistent with reduced reliance on executive control when visuo-tactile signals were congruent (Mayer et al. 2017, Ehlis et al. 2024). These neural dynamics parallel participants’ reports, which described the onset of integration in State 1–1 and the emerging sense of ownership in State 1–5.

In the beta2 band, the most striking feature was the decrease in DC at State 1–3 under the Async condition, particularly in the L-PMd, R-IPL, and L-dlPFC. Rather than reflecting enhanced engagement, this transient suppression appears to mark the detection of conflict and the destabilization of existing body representations. Beta oscillations are widely associated with maintaining the “status quo” of sensorimotor states (Engel and Fries 2010, Kilavik et al. 2013). Thus, their reduction at State 1–3 suggests that the system temporarily suspends stability when faced with incongruent inputs, allowing for the possibility of recalibration.

In the L-PMd, this decrease was followed by renewed increases at State 1–5 and State 2, consistent with a biphasic adaptation process: initial downregulation signaling mismatch detection, and subsequent recovery supporting the incorporation and stabilization of new body representations (Ostry and Gribble 2016, Noppeney 2021). Similarly, the suppression in the R-IPL and L-dlPFC at State 1–3 likely reflects a breakdown of visuospatial integration and accumulation of prediction errors, with the rebound at State 2′ indicating the engagement of top-down control to consolidate a non-illusory outcome (Seth et al. 2012, Chancel et al. 2022).

From this perspective, the beta2-band findings highlight the critical role of transient decreases, rather than sustained increases, in signaling perceptive dissonance and triggering adaptive reorganization. Importantly, this dynamic complements the theta-band effects observed in the PMd: whereas theta oscillations support flexible integration toward illusory states, beta2 oscillations register moments of destabilization that precede either recalibration or rejection of the illusion.

Taken together, the theta and beta2 findings suggest a frequency-specific division of labor in the RHI. Theta oscillations facilitate flexible multisensory integration and the progressive reorganization of bodily experience toward the illusory state, whereas beta2 oscillations register moments of destabilization by signaling the breakdown of existing sensorimotor representations in the face of incongruent inputs. Within this framework, the left PMd emerged as a critical hub that bridges these dual demands: in the theta band, it supported the gradual transition from early multisensory integration (State 1–1/1–2) to the emergence (State 1–5) and stabilization (State 2) of ownership, while in the beta2 band, it exhibited a transient reduction at State 1–3 reflecting conflict detection, followed by a rebound that marked adaptive recalibration. Rather than being contradictory, these dynamics reveal complementary mechanisms through which the PMd contributes both to the flexible formation of body ownership and to the resolution of sensory conflict. By capturing these frequency- and state-specific processes, our study extends prior EEG work on the RHI that largely emphasized alpha and gamma activity (Kanayama et al. 2009, Lenggenhager et al. 2011), providing new evidence that mid-range oscillations, particularly theta and beta2, are central to the fine-grained temporal dynamics of body ownership.

### Limitations

Finally, several methodological limitations should be acknowledged. First, because participants were required to speak during the RHI experiment, speech-related artifacts were inevitably present in the EEG recordings. To mitigate this issue, ICA was applied to remove non-neural activity prior to estimating functional connectivity, and lagged coherence was adopted as the connectivity metric. By selectively capturing phase-delayed interactions while ignoring zero-phase correlations, lagged coherence reduces the influence of spatially diffuse components such as ocular or muscular activity (Pascual-Marqui 2007). These precautions suggest that speech artifacts did not substantially affect the present results. Second, because the interview analysis relied on participants’ natural speech, the amount of subjective reporting varied across states In the EEG classification analysis, no distinctive neural network pattern was identified for State 1–4, despite the fact that this category was represented by a sufficient number of experiential instances in the interview data. One possible explanation is that State 1–4, defined as Comparative Attention, encompassed relatively diverse forms of attentional comparison across sensory modalities or body locations, and therefore may not have corresponded to a single homogeneous neural pattern. Future studies will be needed to determine whether this phenomenologically identified category is associated with distinguishable neural signatures. Third, although the GNN was trained on multiple epoch-derived connectivity matrices, these samples were nested within a modest number of participants (*N* = 16). As a result, the learned representations may have been influenced in part by participant-specific structure, and the generalizability of the present findings should be interpreted with caution. Future studies with larger cohorts will be necessary to evaluate the robustness and reproducibility of the observed network patterns. An additional methodological consideration concerns ROI selection. The present ROI set was defined in a hypothesis-driven manner to sample a distributed network relevant not only to body ownership, but also to multisensory integration and self-related processing more broadly, rather than a minimal ownership-specific core. Importantly, changing the ROI set would not directly alter the connectivity values between already-defined ROI pairs. However, it could change the overall pool of candidate connections and thereby influence normalized DC after top-20% edge thresholding. Future work should therefore examine the robustness of the present findings under alternative ROI definitions informed by recent meta-analytic evidence.

## Conclusion

In summary, this study combined phenomenological interviews with graph-based EEG analyses to examine the dynamic processes underlying the RHI. By aligning subjective states with EEG network states via GW optimal transport, we showed that theta and beta2 oscillations make distinct yet complementary contributions to body ownership: theta facilitates flexible integration and reorganization toward the illusion, while beta2 helps preserve stability against incongruent sensory information. ROI-specific analyses further revealed that premotor, insular, prefrontal, and parietal regions reorganize in a state- and condition-dependent manner, underscoring that body ownership arises from the interplay of multisensory integration, self-referential processes, and conflict resolution. More broadly, these findings suggest that bodily selfhood may not emerge as a simple binary achievement, but rather as a dynamic process unfolding through intermediate experiential and neural transitions. By showing that these transitions can be investigated through the correspondence between phenomenologically defined states and EEG network dynamics, the present study provides a neurophenomenological framework for examining the temporal constitution of the embodied self.

Beyond these empirical contributions, the study demonstrates the value of neurophenomenology as a methodological framework. By systematically combining fine-grained first-person reports with computational models of brain network dynamics, we provide a principled means of bridging subjective experience and neural measures. This approach not only refines current accounts of body ownership but also offers a transferable template for investigating other conscious phenomena, including agency, self–other boundaries, and the narrative self. Future neurophenomenological research may benefit from adopting similar mappings based on state transitions to capture the evolving structure of lived experience in relation to large-scale neural dynamics.

## Supplementary Material

Supplementary_clean_niag040

## Data Availability

The data that support the findings of this study are available from the corresponding author upon reasonable request.
