# Supplementary material for: Bridging subjective and neural state transitions in the rubber hand illusion: a neurophenomenological study
Source: Neurosci Conscious. 2026 Jul 29;2026(1):niag040. doi: 10.1093/nc/niag040 (PMC13417469; doi:10.1093/nc/niag040)
Supplement: Supplementary_clean_niag040 [file supplementary_clean_niag040.docx]

**Supplementary Information**

**Interview training session**

To familiarize participants with the elicitation interview technique, we conducted a brief training session (<10 min) using riddles. The purpose was to encourage participants to practice providing rich, detailed descriptions during the subsequent RHI induction. In this session, participants were asked to report what they were thinking at each moment, and the experimenter posed follow-up questions based on their responses to elicit further elaboration. For example, if a participant said, “I recalled place names” (because the riddle’s answer was a place name), the experimenter might ask, “How did you recall them? Did you visualize a map of Japan in your mind?” This training helped prepare participants to articulate their experiences with greater precision and detail during the main task.

**Selection of ROI**

The premotor cortex has been consistently identified as a core structure involved in the experience of illusory hand ownership and shifts in perceived limb position (Blanke, 2012). In particular, it has been repeatedly implicated as a key cortical hub supporting visuo-tactile integration and the sense of body ownership, with robust and consistent activation across RHI paradigms (e.g., Bekrater-Bodmann et al., 2012, 2014; Ehrsson et al., 2004; Isayama et al., 2019; Peviani et al., 2018; Zeller et al., 2015, 2016).

We focused on two functionally distinct premotor subregions: the bilateral ventral premotor cortex (PMv; BAs 44 and 45; ROIs 1/13) and the bilateral lateral dorsal premotor cortex (PMd; BAs 6 and 8; ROIs 2/14). These regions have been shown to engage in coordinated activity with parietal cortices during RHI induction, supporting a frontoparietal network underlying altered body representation (Gentile et al., 2013; Limanowski & Blankenburg, 2016). Within this network, we included two parietal ROIs: the bilateral superior parietal lobule (SPL; BAs 5 and 7; ROIs 3/15), which has been implicated in proprioceptive and somatosensory integration relevant to bodily awareness (Isayama et al., 2019; Castro et al., 2023; Tsakiris et al., 2007), and the bilateral inferior parietal lobule (IPL; BAs 39 and 40; ROIs 4/16), encompassing the supramarginal and angular gyri, which has been associated with self-body recognition and multisensory processing (Isayama et al., 2019; Zeller et al., 2016; Ismail & Shimada, 2019; Kammers et al., 2009).

We also included the bilateral primary motor (M1; BA 4) and somatosensory cortices (S1; BAs 1–3; ROIs 5/17), which have been shown to undergo task-dependent modulation during RHI tasks, reflecting changes in body representation (Isayama et al., 2019; Della Gatta et al., 2016; Fossataro et al., 2018; Karabanov et al., 2017; Reader et al., 2023; Shibuya et al., 2018, 2019; Zeller et al., 2015, 2016; Schmalzl et al., 2014; Frey et al., 2020). Additionally, the bilateral visual cortex (VC: BAs 17, 18, and 19; ROIs 6/18) (Zeller et al., 2015; Schmalzl et al., 2014; Lee & Chae, 2016; Limanowski et al., 2014; Rossi Sebastiano et al., 2024; Wawrzyniak et al., 2018) and the bilateral insula (Ins: BA 13; ROIs 7/19) (Castro et al., 2023; Limanowski et al., 2014; Tsakiris et al., 2007; Wawrzyniak et al., 2018) were included, given their established roles in visual processing and interoceptive integration during the RHI. The posterior cingulate (PCC; BAs 23, 31; ROIs 8/20) activity reflected integration of self-location and body ownership (Guterstam et al., 2015). The superior temporal gyrus (STG; BAs 21, 22; ROIs 9/21), which has been implicated in body agnosia when lesioned (Moro et al., 2008), was also included due to its potential relevance to disturbed body representation.

The default mode network (DMN), a large-scale system active at rest and involved in introspective processes (Andrews-Hanna et al., 2010), was defined in terms of its midline core and medial temporal subsystem. The midline core included the medial prefrontal cortex (mPFC; BAs 32, 24, and 25 (including anterior cingulate cortex); ROIs 10/22), as well as the posterior cingulate cortex. The medial temporal subsystem comprised the medial temporal lobe (MTL; BAs 28, 34, 35, and 36; ROIs 11/23), which supports memory-based self-representation (Buckner et al., 2008).

Finally, we defined the dorsolateral prefrontal cortex (dlPFC; BAs 9, 10, and 46; ROIs 12/24), which has been associated with introspective monitoring (Miyamoto et al., 2017) and confidence estimation in metacognitive tasks (Shekhar & Rahnev, 2018).

It should be noted that the present ROI set was not intended as a minimal or definitive anatomical model of body ownership derived exclusively from meta-analytic convergence. Rather, it was defined in a hypothesis-driven manner to sample a distributed network of regions discussed in relation to body ownership, multisensory integration, and self-related processing, because the aim of the present study was to examine the broader experiential and neural dynamics through which bodily selfhood emerges. In this respect, the present ROI definition should be understood as theory-driven rather than as a direct attempt to reproduce a single meta-analytic parcellation, and it therefore includes regions that may not be retained under a stricter ROI definition constrained by meta-analytic evidence (e.g., Grivaz et al., 2017; Brunello et al., 2025).

Importantly, altering the ROI set would not change the connectivity values among already-defined ROI pairs themselves, because those pairwise estimates are computed independently of the total number of ROIs. However, because graph construction in the present study retained only the top 20% of candidate edges, changing the ROI set would modify the pool of candidate connections and could consequently affect which edges were retained in the final graph. As a result, graph-theoretical measures such as normalized degree centrality may vary under alternative ROI definitions. Future work should therefore test the robustness of the present findings using alternative ROI sets and parcellation schemes, including sets more strictly constrained by meta-analytic evidence.

**Descriptions of Subjective States**

State 1, *Multisensory Integration,* comprises five substates that lead either to the illusory state (State 2) or the non-illusory state (State 2′). Below, we present explanations and representative participant quotations for each substate, as well as for the illusory and non-illusory states. In each quotation, participants are referenced by their ID and condition (e.g., Sub12-Sync). Key excerpts are italicized for emphasis.

*State1: Multisensory integration*

In this state, participants observed the paintbrush stroking the rubber hand while simultaneously feeling strokes on their own hand, leading to either a clear correspondence or a perceived mismatch between the stimuli. We identified five substates within this process, which were observed across both the synchronous and asynchronous conditions.

*State 1-1: Perception of tactile stimulation.*

This substate captures participants’ direct descriptions of the tactile qualities of the brush strokes, such as their movement or texture.

“*It's like being stroked with the tip of a brush.*” (Sub12-Sync)

*State1-2: Visuo-tactile congruence*

This substate, observed only under the synchronous condition, reflects participants’ awareness that the brush strokes on the rubber hand and their own hand occurred simultaneously, with matching timing and location.

“*Right now it kind of feels like the model hand is being touched, and my right hand is being stroked at the same timing and in the same spot.*” (Sub06-Sync)

*State 1-2’: visuo-tactile incongruence.*

This substate, observed only under the asynchronous condition, reflects participants’ awareness of temporal mismatches between the brush strokes seen on the rubber hand and the tactile sensations felt on their own hand.

“When I look at it, *the timing of when the model hand is being stroked and the timing when I actually feel it on my hand are different*, so I can tell the timing is off.” (Sub10-Async)

*State 1-3: Perceptive dissonance.*

This substate reflects a subtle sense of strangeness or change toward the rubber hand. It resembles the “violation of expectations” phase in Valenzuela-Moguillansky’s structure, but here it includes a dissonance arising from discrepancies between visual, tactile, and hand-position inputs, as well as a vague sense of surprise at changes in the appearance of the rubber hand and the sensations experienced when it is touched— without a clear sense of ownership.

“I can’t fully believe the hand I see in front of me is my own, but every time the brush is about to touch it, I kind of tense up for a moment—*I think, ‘it’s going to touch me,’ but then it doesn’t,* so *I feel a bit restless*. At the same time, it still looks a bit too beautiful, so I know it’s not really my hand.” (Sub03-Async)

“When the hand I’m looking at is being stroked, but I don’t actually feel the sensation, *it feels a bit odd to watch*.” (Sub12-Async)

*State 1-4: Comparative attention.*

This substate reflects higher-order cognitive processes, such as shifts of attention and active comparisons across situations. It resembles and extends the “concentration on the rubber hand” phase in Valenzuela-Moguillansky’s structure, involving alternating focus between the rubber hand and one’s own hand, or comparing the present experience with past or typical ones, in order to validate perception. Unlike State 1-3, which reflects more passive and uncertain impressions, State 1-4 is characterized by deliberate processes such as predicting the timing of tactile sensations or recalling prior tactile experiences.

“*If I focus on the model hand, it kind of looks like my own hand, but when I shift my attention to my right hand, I still feel a bit of a mismatch*, *like the location feels off.*” (Sub10-Sync)

“*When I’m not being stroked, I kind of feel like this hand might be mine. But when I’m stroked, there’s a mismatch between what I see and what I feel*, *so the moment I’m stroked, I feel like, oh, this is actually something separate from me.*” (Sub10-Async)

*State 1-5: Beginning of the incorporation of the rubber hand.*

This substate includes descriptions in which participants began to perceive the rubber hand as part of their own body. Compared to State 1-2, participants reported a stronger association between visual and tactile inputs, accompanied by a growing sense of visual familiarity. Some described the rubber hand as a projection or reflection of their hidden real hand, suggesting resolution of the spatial conflict regarding its location. Others reported a slight sense of agency, feeling as though they might move the rubber hand. Unlike State 1-4, where ownership appeared only in specific moments, reports in State 1-5 conveyed a more continuous—though still tentative—sense of ownership and/or confusion regarding the location of one’s own hand or tactile sensations.

“At first, I thought the position of my hand was a bit further out than what I was actually seeing, but now, *I’m not really sure about the sense of its location, and the connection between what I see and the feeling of being touched has kind of taken priority*—that’s the feeling I get.” (Sub03-Sync)

“*It does feel a bit more like my own hand than at the very start. Like, the skin color kind of looks like my real hand.*” (Sub11-Async)

“*It feels like my hand is floating* […] *I feel like the hand I can’t see is moving.* Compared to the beginning, *I’ve lost track of where my hand is.*” (Sub01-Sync)

“Somehow, since it’s inside the box, I can’t see it—I mean, I can’t see my own right hand—but with the model’s right hand… how should I put it… *it kind of feels like my own right hand inside the box is being shown as an image on the screen through the model’s hand.*” (Sub06-Sync)

“*I kind of feel like the hand I’m looking at might start to move*—more so than at the beginning, I think.” (Sub08-Sync) (Unlike the example statements in the RHI category, there is low confidence regarding the ability to move.)

*State 2: Illusory state*

This state refers to the full emergence of the illusion. Compared with State 1-5, participants’ descriptions conveyed greater confidence and more pronounced characteristics. Four types of illusory experiences were identified:

**Typical Rubber Hand Illusion (RHI):** Clear ownership of the rubber hand, alignment of its location with the real hand, and sometimes a sense of agency.

“*It feels like the sensation of my real hand and the hand I see are the same. It’s like my hand has come right in front of me.* I don’t know… *the hand in front of me feels like it’s my own.* Compared to when I started, *I can’t really sense my real hand properly anymore.*” (Sub01-Sync)

“At the beginning, I felt a difference between the model hand and my own hand, but *now it feels like there’s only one sensation*.”

(Experimenter) “And where do you feel that one sensation?”

“Where… do you mean, like, *in the middle finger*?”

(Experimenter) “The middle finger… which middle finger?”

“Ah—uh, the model hand’s? *The one in front of me*. […] *It already feels like my own hand.*” (Sub14-Sync)

“It's not really ticklish, and it's not a reflex, but *I feel like I want to move it somehow.*” (Sub08-Sync) (Unlike the example statements in State 1‑5, there is an increased sense of agency or desire to move the hand oneself.)

**Double Hand Illusion (DHI):** A simultaneous sense of owning both the rubber hand and one’s own hand.

“I feel like the fake hand might be my own hand, but I can still feel my real hand, and… somehow *it feels like I have two hands*.” (Sub02-Sync)

**Phantom Tactile Sensation (PTS):** Feeling touch on the real hand when only the rubber hand was stroked, observed exclusively in the asynchronous condition.

“Well… *when the fake hand was being touched, my right hand, which shouldn’t have been touched—my real right hand—felt like it was being touched*.” (Sub05-Async)

**Continuous Weak Illusion (CWI):** A prolonged, low-intensity sense of ownership resembling State 1-5 but persisting for more than three minutes, interpreted as a stable form of the illusion.

*State 2’: non-illusory state.*

This state corresponds to the absence of illusion, typically observed in the latter part of the induction. Participants reported no change in the perceived location of their real hand and no sense of ownership toward the rubber hand.

“*I can’t really feel like the fake hand is my own.*” (Sub04-Async)

“*I still feel the sensation in my real hand*, so I don’t think it’s changed much.” (Sub14-Async)

The time course of participants’ subjective state transitions in the Sync and Async conditions are shown in Supplementary Figure 1.


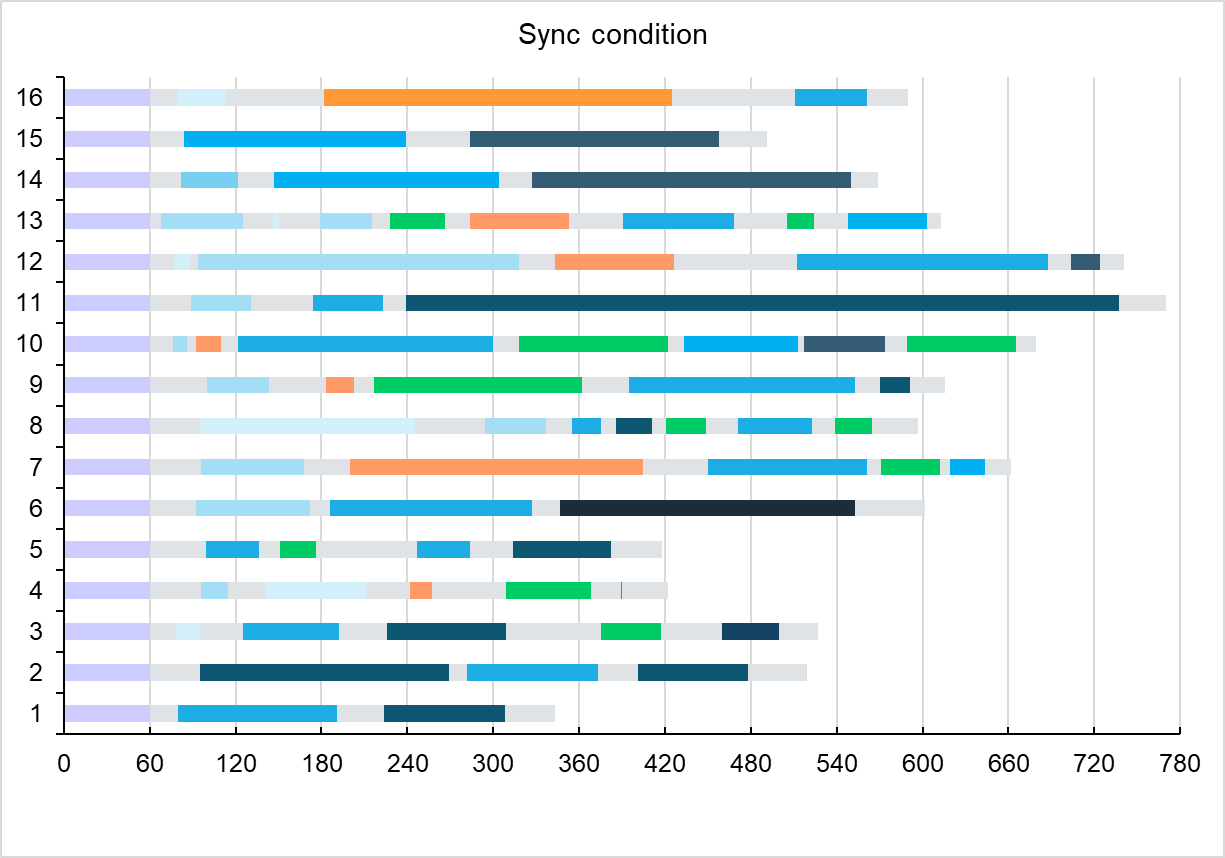


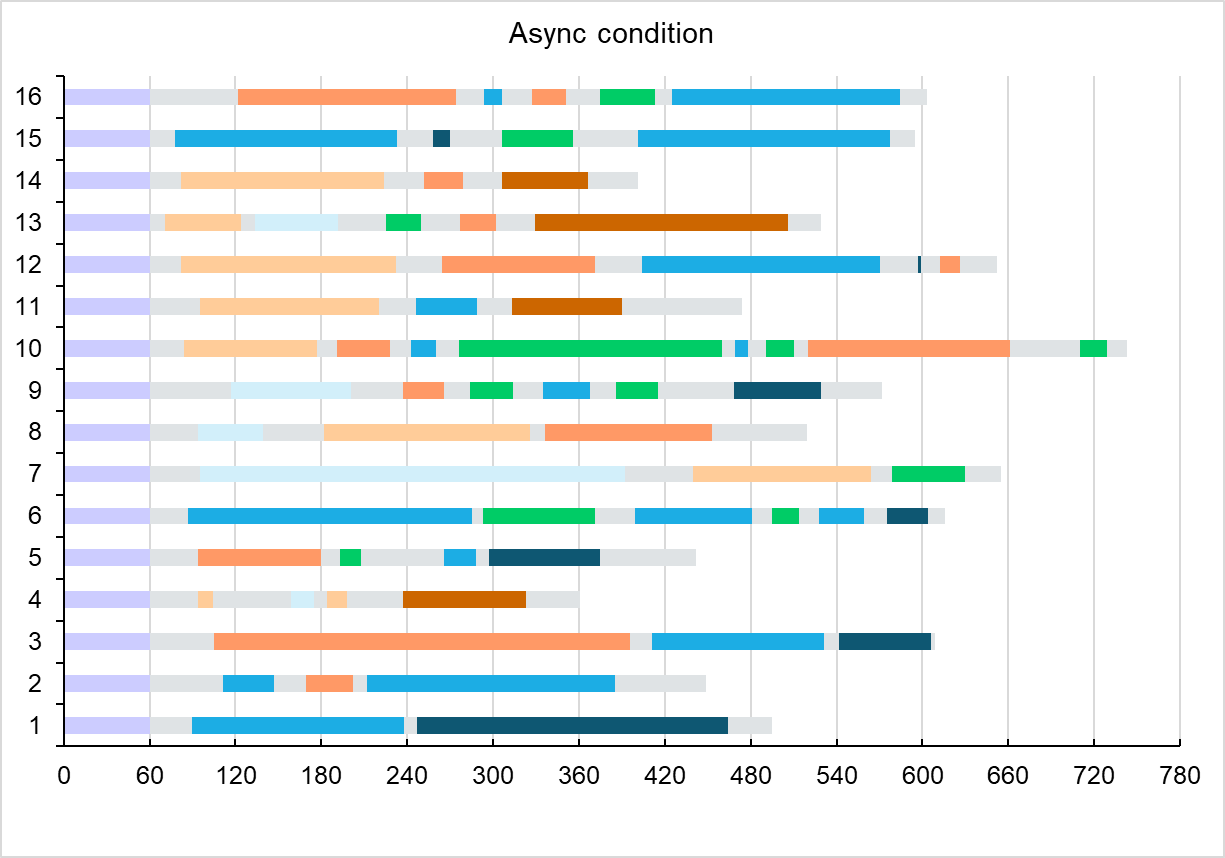


**S. Figure 1. Time course of subjective state transitions.** Panels show state transitions for the Sync (top) and Async (bottom) conditions. State durations were defined based on timing of participants’ experiential descriptions. Consecutive descriptions assigned to the same category were treated as a single segment spanning from the first to the last corresponding report, including intervening silence and experimenter prompts. Periods without classification were labeled as transitions.

**Pairwise GW distances across frequency bands**

To identify the frequency bands with the strongest state divergence, we computed the average functional connectivity matrix for each mapped state and then calculated the mean GW distance across all state pairs within each band (S. Figure 2). A Wilcoxon signed-rank test was then performed to compare the mean GW distances across all frequency bands, and the resulting p-values were corrected for multiple comparisons using the Bonferroni method. As a result, the Theta and Beta2 bands showed significantly smaller GW distances compared to the other bands. The complete results of the pairwise comparisons are summarized in S. Table 1.


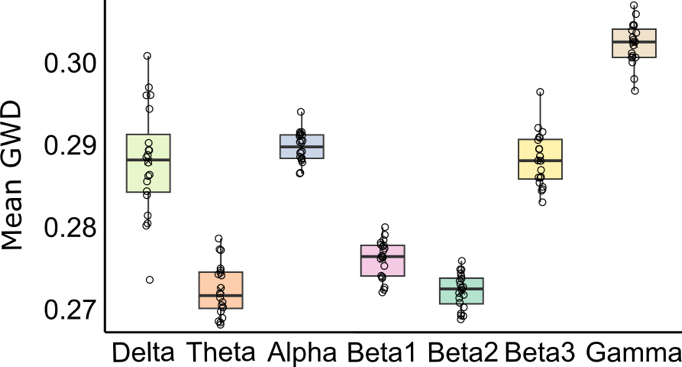


**S. Figure 2. Mean GW distance across all state pairs.** The box plots illustrate the interquartile range, the horizontal line denotes the median, and whiskers represent the minimum and maximum values within 1.5 × the interquartile range. The mean values of each state are represented as crosses and individual data points for each run of the mapping procedure are represented as circles.

**S. Table 1.** Results of pairwise Wilcoxon signed-rank tests

| **Comparison** | **W** | **p.adj** |
| --- | --- | --- |
| Delta v.s. Theta | 0 | < 0.0001 |
| Delta v.s. Alpha | 72 | 1 |
| Delta v.s. Beta1 | 1 | < 0.0001 |
| Delta v.s. Beta2 | 0 | < 0.0001 |
| Delta v.s. Beta3 | 98 | 1 |
| Delta v.s. Gamma | 0 | < 0.0001 |
| Theta v.s. Alpha | 0 | < 0.0001 |
| Theta v.s. Beta1 | 15 | < 0.01 |
| Theta v.s. Beta2 | 97 | 1 |
| Theta v.s. Beta3 | 0 | < 0.0001 |
| Theta v.s. Gamma | 0 | < 0.0001 |
| Alpha v.s. Beta1 | 0 | < 0.0001 |
| Alpha v.s. Beta2 | 0 | < 0.0001 |
| Alpha v.s. Beta3 | 59 | 1 |
| Alpha v.s. Gamma | 0 | < 0.0001 |
| Beta1 v.s. Beta2 | 11 | < 0.01 |
| Beta1 v.s. Beta3 | 0 | < 0.0001 |
| Beta1 v.s. Gamma | 0 | < 0.0001 |
| Beta2 v.s. Beta3 | 0 | < 0.0001 |
| Beta2 v.s. Gamma | 0 | < 0.0001 |
| Beta3 v.s. Gamma | 0 | < 0.0001 |

Adjusted p values (p.adj) are reported using Bonferroni correction.

**References**

Andrews-Hanna, J. R., Reidler, J. S., Sepulcre, J., Poulin, R., & Buckner, R. L. (2010). Functional-Anatomic Fractionation of the Brain’s Default Network. *Neuron*, *65*(4), 550–562. <https://doi.org/10.1016/j.neuron.2010.02.005>

Bekrater-Bodmann, R., Foell, J., Diers, M., & Flor, H. (2012). The perceptual and neuronal stability of the rubber hand illusion across contexts and over time. *Brain Research*, *1452*, 130–139. <https://doi.org/10.1016/j.brainres.2012.03.001>

Bekrater-Bodmann, R., Foell, J., Diers, M., Kamping, S., Rance, M., Kirsch, P., Trojan, J., Fuchs, X., Bach, F., Çakmak, H. K., Maaß, H., & Flor, H. (2014). The Importance of Synchrony and Temporal Order of Visual and Tactile Input for Illusory Limb Ownership Experiences – An fMRI Study Applying Virtual Reality. *PLoS ONE*, *9*(1), e87013. <https://doi.org/10.1371/journal.pone.0087013>

Blanke, O. (2012). Multisensory brain mechanisms of bodily self-consciousness. *Nature Reviews Neuroscience*, *13*(8), 556–571. <https://doi.org/10.1038/nrn3292>

Brunello, N., Diana, L., Sritharan, J., Glisic, M., Nef, T., Verma, R. K., & Zito, G. A. (2025). A systematic review and meta-analysis on the neural correlates of bodily self-consciousness. *Neuroscience & Biobehavioral Reviews*, *179*, 106420. <https://doi.org/10.1016/j.neubiorev.2025.106420>

Buckner, R. L., Andrews‐Hanna, J. R., & Schacter, D. L. (2008). *The Brain’s Default Network*: *Anatomy, Function, and Relevance to Disease*. *Annals of the New York Academy of Sciences*, *1124*(1), 1–38. <https://doi.org/10.1196/annals.1440.011>

Castro, F., Lenggenhager, B., Zeller, D., Pellegrino, G., D’Alonzo, M., & Di Pino, G. (2023). From rubber hands to neuroprosthetics: Neural correlates of embodiment. *Neuroscience & Biobehavioral Reviews*, *153*, 105351. <https://doi.org/10.1016/j.neubiorev.2023.105351>

Della Gatta, F., Garbarini, F., Puglisi, G., Leonetti, A., Berti, A., & Borroni, P. (2016). Decreased motor cortex excitability mirrors own hand disembodiment during the rubber hand illusion. *eLife*, *5*, e14972. <https://doi.org/10.7554/eLife.14972>

Ehrsson, H. H., Spence, C., & Passingham, R. E. (2004). That’s My Hand! Activity in Premotor Cortex Reflects Feeling of Ownership of a Limb. *Science*, *305*(5685), 875–877. <https://doi.org/10.1126/science.1097011>

Fossataro, C., Bruno, V., Giurgola, S., Bolognini, N., & Garbarini, F. (2018). Losing my hand. Body ownership attenuation after virtual lesion of the primary motor cortex. *European Journal of Neuroscience*, *48*(6), 2272–2287. <https://doi.org/10.1111/ejn.14116>

Frey, V. N., Butz, K., Zimmermann, G., Kunz, A., Höller, Y., Golaszewski, S., Trinka, E., & Nardone, R. (2020). Effects of Rubber Hand Illusion and Excitatory Theta Burst Stimulation on Tactile Sensation: A Pilot Study. *Neural Plasticity*, *2020*, 1–8. <https://doi.org/10.1155/2020/3069639>

Gentile, G., Guterstam, A., Brozzoli, C., & Ehrsson, H. H. (2013). Disintegration of Multisensory Signals from the Real Hand Reduces Default Limb Self-Attribution: An fMRI Study. *The Journal of Neuroscience*, *33*(33), 13350–13366. <https://doi.org/10.1523/JNEUROSCI.1363-13.2013>

Grivaz, P., Blanke, O., & Serino, A. (2017). Common and distinct brain regions processing multisensory bodily signals for peripersonal space and body ownership. *NeuroImage*, *147*, 602–618. <https://doi.org/10.1016/j.neuroimage.2016.12.052>

Guterstam, A., Abdulkarim, Z., & Ehrsson, H. H. (2015). Illusory ownership of an invisible body reduces autonomic and subjective social anxiety responses. *Scientific Reports*, *5*(1), 9831. <https://doi.org/10.1038/srep09831>

Isayama, R., Vesia, M., Jegatheeswaran, G., Elahi, B., Gunraj, C. A., Cardinali, L., Farnè, A., & Chen, R. (2019). Rubber hand illusion modulates the influences of somatosensory and parietal inputs to the motor cortex. *Journal of Neurophysiology*, *121*(2), 563–573. <https://doi.org/10.1152/jn.00345.2018>

Ismail, M. A. F. B., & Shimada, S. (2019). Activity of the inferior parietal cortex is modulated by visual feedback delay in the robot hand illusion. *Scientific Reports*, *9*(1), 10030. <https://doi.org/10.1038/s41598-019-46527-8>

Kammers, M. P. M., Verhagen, L., Dijkerman, H. C., Hogendoorn, H., De Vignemont, F., & Schutter, D. J. L. G. (2009). Is This Hand for Real? Attenuation of the Rubber Hand Illusion by Transcranial Magnetic Stimulation over the Inferior Parietal Lobule. *Journal of Cognitive Neuroscience*, *21*(7), 1311–1320. <https://doi.org/10.1162/jocn.2009.21095>

Karabanov, A. N., Ritterband‐Rosenbaum, A., Christensen, M. S., Siebner, H. R., & Nielsen, J. B. (2017). Modulation of fronto‐parietal connections during the rubber hand illusion. *European Journal of Neuroscience*, *45*(7), 964–974. <https://doi.org/10.1111/ejn.13538>

Lee, I.-S., & Chae, Y. (2016). Neural Network Underlying Recovery from Disowned Bodily States Induced by the Rubber Hand Illusion. *Neural Plasticity*, *2016*, 1–9. <https://doi.org/10.1155/2016/8307175>

Limanowski, J., & Blankenburg, F. (2016). Integration of Visual and Proprioceptive Limb Position Information in Human Posterior Parietal, Premotor, and Extrastriate Cortex. *The Journal of Neuroscience*, *36*(9), 2582–2589. <https://doi.org/10.1523/JNEUROSCI.3987-15.2016>

Limanowski, J., Lutti, A., & Blankenburg, F. (2014). The extrastriate body area is involved in illusory limb ownership. *NeuroImage*, *86*, 514–524. <https://doi.org/10.1016/j.neuroimage.2013.10.035>

Miyamoto, K., Osada, T., Setsuie, R., Takeda, M., Tamura, K., Adachi, Y., & Miyashita, Y. (2017). Causal neural network of metamemory for retrospection in primates. *Science*, *355*(6321), 188–193. <https://doi.org/10.1126/science.aal0162>

Moro, V., Urgesi, C., Pernigo, S., Lanteri, P., Pazzaglia, M., & Aglioti, S. M. (2008). The Neural Basis of Body Form and Body Action Agnosia. *Neuron*, *60*(2), 235–246. <https://doi.org/10.1016/j.neuron.2008.09.022>

Peviani, V., Magnani, F. G., Ciricugno, A., Vecchi, T., & Bottini, G. (2018). Rubber Hand Illusion survives Ventral Premotor area inhibition: A rTMS study. *Neuropsychologia*, *120*, 18–24. <https://doi.org/10.1016/j.neuropsychologia.2018.09.017>

Reader, A. T., Coppi, S., Trifonova, V. S., & Ehrsson, H. H. (2023). No reduction in motor‐evoked potential amplitude during the rubber hand illusion. *Brain and Behavior*, *13*(10), e3211. <https://doi.org/10.1002/brb3.3211>

Rossi Sebastiano, A., Poles, K., Gualtiero, S., Romeo, M., Galigani, M., Bruno, V., Fossataro, C., & Garbarini, F. (2024). Balancing the Senses: Electrophysiological Responses Reveal the Interplay between Somatosensory and Visual Processing During Body-Related Multisensory Conflict. *The Journal of Neuroscience*, *44*(19), e1397232024. <https://doi.org/10.1523/JNEUROSCI.1397-23.2024>

Schmalzl, L., Kalckert, A., Ragnö, C., & Ehrsson, H. H. (2014). Neural correlates of the rubber hand illusion in amputees: A report of two cases. *Neurocase*, *20*(4), 407–420. <https://doi.org/10.1080/13554794.2013.791861>

Shekhar, M., & Rahnev, D. (2018). Distinguishing the Roles of Dorsolateral and Anterior PFC in Visual Metacognition. *The Journal of Neuroscience*, *38*(22), 5078–5087. <https://doi.org/10.1523/JNEUROSCI.3484-17.2018>

Shibuya, S., Unenaka, S., Zama, T., Shimada, S., & Ohki, Y. (2018). Spontaneous imitative movements induced by an illusory embodied fake hand. *Neuropsychologia*, *111*, 77–84. <https://doi.org/10.1016/j.neuropsychologia.2018.01.023>

Shibuya, S., Unenaka, S., Zama, T., Shimada, S., & Ohki, Y. (2019). Sensorimotor and Posterior Brain Activations During the Observation of Illusory Embodied Fake Hand Movement. *Frontiers in Human Neuroscience*, *13*, 367. <https://doi.org/10.3389/fnhum.2019.00367>

Tsakiris, M., Hesse, M. D., Boy, C., Haggard, P., & Fink, G. R. (2007). Neural Signatures of Body Ownership: A Sensory Network for Bodily Self-Consciousness. *Cerebral Cortex*, *17*(10), 2235–2244. <https://doi.org/10.1093/cercor/bhl131>

Valenzuela Moguillansky, C., O’Regan, J. K., & Petitmengin, C. (2013). Exploring the subjective experience of the “rubber hand” illusion. *Frontiers in Human Neuroscience*, *7*. <https://doi.org/10.3389/fnhum.2013.00659>

Wawrzyniak, M., Klingbeil, J., Zeller, D., Saur, D., & Classen, J. (2018). The neuronal network involved in self-attribution of an artificial hand: A lesion network-symptom-mapping study. *NeuroImage*, *166*, 317–324. <https://doi.org/10.1016/j.neuroimage.2017.11.011>

Zeller, D., Friston, K. J., & Classen, J. (2016). Dynamic causal modeling of touch-evoked potentials in the rubber hand illusion. *NeuroImage*, *138*, 266–273. <https://doi.org/10.1016/j.neuroimage.2016.05.065>

Zeller, D., Litvak, V., Friston, K. J., & Classen, J. (2015). Sensory Processing and the Rubber Hand Illusion—An Evoked Potentials Study. *Journal of Cognitive Neuroscience*, *27*(3), 573–582. <https://doi.org/10.1162/jocn_a_00705>
